# Dehydrodiisoeugenol targets the PLK1-p53 axis to inhibit breast cancer cell cycle

**DOI:** 10.3389/fphar.2025.1545498

**Published:** 2025-02-28

**Authors:** Lin Li, Yifan Zheng, Yongxia Yang, Senlin Shi, Shangjie Liu, Keying Huang, Luonan Qiu, Rongxin Zhang, Wenbin Huang, Yin Leng

**Affiliations:** ^1^ Department of General Surgery Ⅱ, The First Affiliated Hospital of Guangdong Pharmaceutical University, Guangzhou, Guangdong, China; ^2^ College of Medical Information Engineering, Guangdong Pharmaceutical University, Guangzhou, China; ^3^ Guangdong Provincial Key Laboratory for Biotechnology Drug Candidates, Institute of Basic Medical Sciences and Department of Biotechnology, School of Life Sciences and Biopharmaceutics, Guangdong Pharmaceutical University, Guangzhou, China; ^4^ Department of Hepatobiliary Surgery Ⅱ, Zhujiang Hospital, Southern Medical University, Guangzhou, China

**Keywords:** breast cancer, dehydrodiisoeugenol, cell cycle, PLK1, P53

## Abstract

**Introduction:**

There are about 2,300,000 new cases of breast cancer worldwide each year. Breast cancer has become the first most common cancer in the world and the leading cause of death among women. At the same time, chemotherapy resistance in patients with advanced breast cancer is still a serious challenge. Alpinia Katsumadai Hayata (AKH), as a traditional Chinese herbal medicine, has a wide range of pharmacological activities. Related studies have found that many compounds in AKH have anti-breast cancer activity. However, it is still worth exploring which component is the main active component of AKH in inhibiting breast cancer and its mechanism of action.

**Methods:**

In this study, dehydrodiisoeugenol (DHIE) was screened as the main active ingredient of AKH against breast cancer based on LC-MS combined with drug similarity and disease enrichment analysis. WGCNA, network pharmacology, molecular docking, transcriptome sequencing analysis, immune infiltration analysis and single-cell sequencing were used to explore the mechanism of DHIE on breast cancer. CCK-8, flow cytometry and Western blot were used to verify the results *in vitro*. The efficacy of the drugs was verified *in vivo* by constructing a subcutaneous tumor-bearing mouse model.

**Results:**

Our research showed that DHIE and breast cancer enriched core gene targets mainly act on epithelial cells in breast cancer tissues and significantly inhibit the growth of breast cancer by affecting the PLK1-p53 signaling axis to arrest the breast cancer cell cycle at G0/G1 phase. Further analysis showed that although DHIE had opposite regulatory effects on different isoforms of p53 in different types of breast cancer cells, they eventually caused cell cycle arrest. In addition, *in vivo* studies showed that DHIE reduced tumor burden, significantly reduced the infiltration level of tumor proliferation-related marker Ki-67, and inhibited the expression of PLK1 in the mouse model, which was further enhanced when combined with DOX.

**Discussion:**

Collectively, our study suggests that DHIE in AHK may eventually induce cell cycle arrest and inhibit breast cancer growth by regulating the PLK1-p53 signaling axis, which may provide a new therapeutic strategy for breast cancer. However, the specific mechanisms by which DHIE regulates p53 in different subtypes of breast cancer and the advantages of chemotherapeutic combinations compared with other drugs are still worth exploring.

## Highlights

1. Targeted Anticancer Mechanism of DHIE: Our study uncovers a novel mechanism by which Dehydrodiisoeugenol (DHIE) inhibits cancer cell proliferation by blocking the breast cancer cell cycle through the PLK1-p53 signaling axis.
2. Synergistic Effect with Chemotherapy: Our research reveals that combining Dehydrodiisoeugenol (DHIE) with the chemotherapeutic drug DOX significantly enhances the survival rates and inhibits tumor progression in a breast cancer mouse model.
3. Molecular Docking and Disease Network Analysis: Utilizing molecular docking technology and network pharmacology methods, the study verifies the interaction between DHIE and breast cancer-related targets, providing a scientific basis for DHIE’s potential as a treatment for breast cancer.

## 1 Introduction

Breast cancer originates from the uncontrolled proliferation of breast epithelial cells and is categorized into Luminal A, Luminal B, HER2-enriched, and Basal-like subtypes based on differential molecular expression. These distinct molecular classifications correspond to specific therapeutic strategies and prognostic levels (Waks and Winer, 2019). Breast cancer, as the most common cancer in the female population, has the second highest cancer incidence rate globally, surpassing gastric cancer with 670,000 deaths to become the world’s fourth leading cancer killer, and far surpassing gynecological tumors such as uterine and ovarian cancers (Chohan et al., 2024). On 1 February 2024, the International Agency for Research on Cancer (IARC) of the World Health Organization (WHO) released the most recent data on the global burden of cancer: 2022 Breast cancer is the most commonly occurring cancer among females in the world. With approximately 2.31 million new cases of breast cancer each year globally, it has become the number one cancer in women worldwide and the leading cause of cancer-related deaths in women (Bray et al., 2024).

Despite the significant success of current conventional treatment approaches, including neoadjuvant chemotherapy, surgery, and adjuvant chemotherapy, in achieving a cure rate of up to 90% in early-stage breast cancer and over 50% in intermediate and late stages, the issue of chemotherapy resistance remains a formidable challenge for patients with advanced breast cancer (Herzog and Fuqua, 2022). Meanwhile, doxorubicin, as the first-line drug for treating breast cancer, although it shows excellent efficacy in treating breast cancer, it also has several major problems: it has adverse side effects such as cardiac toxicity, cognitive impairment, myelosuppression, alopecia and hand-foot syndrome (Bews et al., 2024; Desai et al., 2024; Hirakata et al., 2024; Prasher and Sharma, 2024), and its efficacy is not significant for patients with advanced breast cancer, and tumor develops drug resistance (Khan et al., 2024). However, chemical sensitizers or targeted therapies may make breast cancer cells re-sensitize to the effects of chemotherapy drugs. Therefore, screening effective therapeutic agents and reducing drug resistance are of great significance in improving the cure rate of breast cancer. In recent years, the use of herbal medicines for clinical adjuvant treatment of tumors has been increasing worldwide.

Alpinia Katsumadai Hayata (AKH), the nearly mature seeds of Alpinia Katsumadai Hayata, family Zingiberaceae. Modern pharmacological studies have shown that nutmeg has a variety of pharmacological effects such as protection of gastric mucosa, anti-gastric ulcer, anti-inflammatory and anti-tumor (Lee et al., 2012; Nam and Seo, 2012; An et al., 2021; Ashokkumar et al., 2022; An et al., 2022). Some of its components have been shown to have anti-breast cancer effects, such as Alpinetin’s dependence on the ROS/NF-κB/HIF-1α axis to inhibit breast cancer growth (Zhang et al., 2023).

The chemical constituents of AKH are mainly volatile oils, flavonoids, diphenylheptanes, terpenoids, polysaccharides and other components. Studies have shown that the active components isolated from AKH such as sanguinarine, naringenin, and galangin have shown inhibitory effects against breast cancer (Liu et al., 2018; Su et al., 2021; Zhang et al., 2023), but mainly focused on flavonoids and terpenoids (Wang et al., 2008), whereas the anti-breast cancer effects of its diarylheptane compounds and other constituents have not been reported.

Dehydrodiisoeugenol (DHIE) is a neolignan found in more than 17 species of plants, including herbs, fruits and rhizomes. DHIE was first isolated from Myrtus communis in 1973. There is now growing evidence that DHIE has a wide range of biological activities: anti-inflammatory, antioxidant, anticancer and antimicrobial properties (Godínez-Chaparro et al., 2022), supporting the potential of DHIE as a therapeutic agent. However, little research has been conducted on DHIE in the field of anticancer, with only seven relevant reports in the last 10 years, all of them focusing on colorectal cancer and salivary gland tumors. Li et al., 2021 found that DHIE treatment caused the cell cycle of colorectal cancer cells to arrest in the G1/S phase, leading to significant inhibition of cell growth (Li et al., 2021). In addition, DHIE induced strong cellular autophagy, which played a role in the growth inhibition of colorectal cancer cells. Further analysis showed that DHIE also induced endoplasmic reticulum (ER) stress and subsequently stimulated autophagy through activation of the PERK/eIF2α and IRE1α/XBP-1s/CHOP pathways. Atsumi et al., 2000, combining a CCK8 cytotoxicity assay with an assessment of free radical intensity measured by electron spin resonance, found that DHIE significantly inhibited the activity and DNA activity of salivary gland tumor cell lines activity as well as DNA synthesis (Atsumi et al., 2000). In conclusion, there is still a lack of research on DHIE in the field of anticancer and there is a large scope for research progress. A full understanding of the effects and mechanisms of action of DHIE in different tumors will provide new therapeutic strategies for the development of new drugs, especially in the area of drug resistance in breast cancer.

Although many current studies have found that a variety of compounds present within AKH are effective in inhibiting breast cancer progression, however, there is a lack of evidence to demonstrate the active components that play a major role in the inhibition of breast cancer by AKH and their utilization in humans. Therefore, in this study, we screened the active ingredients based on the pharmacogenetic properties of the compounds within AKH in the TCMSP database and combined them with LC-MS for the screening of the main active ingredients, integrating bioinformatics methods such as WGCNA, network pharmacology, transcriptome sequencing analysis, and single-cell sequencing analysis to screen for the targets of active ingredients affecting breast cancer and the related pathways and mechanisms of action. In addition, we will conduct experiments to investigate the pharmacological effects and potential mechanisms of the main active ingredients in AKH on breast cancer *in vivo* and *in vitro*.

## 2 Materials and methods

### 2.1 Data collection

The data set comprised 1081 gene expression matrices from breast cancer tissues and 99 from adjacent normal tissues, sourced from the Cancer Genome Atlas (TCGA) database (https://portal.gdc.cancer.gov/). Additionally, the Gene Expression Omnibus (GEO) database (https://www.ncbi.nlm.nih.gov/geo/) was consulted for further data. The data set GSE180286, comprising five primary breast cancer samples, was obtained from the Gene Expression Omnibus (GEO) database (https://www.ncbi.nlm.nih.gov/geo/). To eliminate batch effects, the data were normalized. The drug targets were identified through the TCMSP (https://old.tcmsp-e.com/tcmsp.php), CTD (https://ctdbase.org/), PharmMapper (https://www.lilab-ecust.cn/pharmmapper/) and Swiss Target Prediction (http://old.swisstargetprediction.ch/help.php) databases.

### 2.2 Prediction of putative targets of AKH

The compounds of Alpinia Katsumadai Hayata (AHK) were sourced from the Traditional Chinese Medicine System Pharmacology Database (TCMSP, https://old.tcmsp-e.com/tcmspsearch.php). The screening criteria for the bioactive compounds were oral bioavailability (OB) ≥40% and drug similarity (DL) ≥0. 20. The selected bioactive compounds were sourced from the PubChem compound database (https://www.ncbi.nlm.nih.gov/p compound) and their structures uploaded to the Swiss Target Prediction database (http://www.swisstargetprediction.ch/) in order to identify specific targets.

### 2.3 LC-MS analysis and disease ontology of compounds in AKH

Accurately weigh 20 mg of AHK lyophilized powder, place in a grinder with two small steel balls and grind at 50 Hz for 5 min, add 100 µL of 80% aqueous methanol, vortex and shake, and let stand on an ice bath for 5 min before centrifuging at 15,000 g for 15 min at 4°C. A certain amount of supernatant was diluted with mass spectrometry-grade water until the methanol content was 53%, and then centrifuged at 15,000 g for 15 min at 4°C, and then supernatant was collected and injected into the LC-MS for analysis. LC-MS analyses were performed using a Vanquish UHPLC system (ThermoFisher, Germany) coupled with an Orbitrap Q Exactive TMHF mass spectrometer or Orbitrap Q Exactive HF-X mass spectrometer (Thermo Fisher, Germany). Samples were injected onto a Hypersil Gold column (100 × 2.1 mm, 1.9 μm) using a 12-min linear gradient at a flow rate of 0.2 mL/min. The eluents for the positive and negative polarity modes were eluent A (0.1% FA in Water) and eluent B (Methanol). The solvent gradient was set as follows: 2% B, 1.5 min, 2%–85% B, 3 min, 85%–100% B, 10 min; 100%–2% B, 10.1 min; 2% B, 12 min. Q ExactiveTM HF mass spectrometer was operated in positive/negative polarity mode with spray voltage of 3.5 kV, capillary temperature of 320°C, sheath gas flow rate of 35 psi and aux gas flow rate of 10 L/min, S-lens RF level of 60, Aux gas heater temperature of 350°C. The raw data files generated by LC-MS were processed using the Compound Discoverer 3.3 (CD3.3, ThermoFisher) to perform peak alignment, peak picking, and quantitation for each metabolite.Statistical analyses were performed using the statistical software R (R version R-3.4.3), Python (Python 2.7.6 version) and CentOS(CentOS release 6.6). Combining the compounds screened according to their pharmacogenicity in the TCMSP database with the compounds collected and relatively quantified in positive and negative modes by LC-MS, we chose three compounds, pinocembrin, dehydrodiisoeugenol and quercetin, for target collection, and used the software packages of “clusterProfiler”, “org.Hs., e.g.,.db,” “enrichplot,” “ggplot2,” and “DOSE” R packages to demonstrate the relevant target-enriched diseases.

### 2.4 Weighted gene co-expression network analysis (WGCNA)

Weighted Correlation Network Analysis (WGCNA) is a computational approach widely used in genomics to construct co-expression networks and identify gene modules with strong functional associations. We used the R package “WGCNA” to perform the WGCNA analysis based on the TCGA-BRCA bulk RNA-seq data. First, an appropriate soft threshold β was calculated to satisfy the criteria for constructing a scale-free network. We then transformed the weighted adjacency matrix into a topological overlap matrix (TOM) and matrix (TOM) and calculated the dissimilarity (dissTOM). To perform gene clustering and module identification, we applied the dynamic tree-cutting approach. To investigate phenotype-module associations, a consensus gene co-expression correlation matrix was generated, with tumor and normal tissues designated as the primary phenotypic comparison groups. Finally, the module with the highest correlation with the breast cancer tumor scores was identified for further analysis. The R2 was set at 0.85.

### 2.5 Network construction and analysis

In order to construct a drug-target-disease network to screen and elucidate the targets of DHIE in breast cancer and its mechanism, the “DESeq2” R package was used to identify differentially expressed genes (DEGs), | Log2FoldChange | DEGs with >1 and p-adjust <0.05 were considered statistically significant, and then combined with modular genes based on WGCNA screening, intersections were taken using Venn online software (http://bioinformatics.psb.ugent.be/webtools/Venn/) to obtain 1258 co-targets as enriched targets for breast cancer. The targets of breast cancer and DHIE were taken for intersection to obtain 24 co-targets. The PPI network was mapped using Cytoscape 3.9.1 and a subset of 13 genes was obtained as core gene clusters in the co-target PPI network according to the MCODE algorithm. The parameters were configured with a node density cutoff of 0.1, node score cutoff of 0.2, k-core of 2, and maximum depth of 100.

### 2.6 Gene ontology and KEGG pathway enrichment analysis

The core gene clusters obtained above were analyzed for GO and KEGG enrichment using the R packages “clusterProfiler”, “org.Hs.e.g.,.db,” “enrichplot,” “ggplot2,” “pathview,” “ggnewscale,” and “DOSE.” GO divides genetic functions into three sections: (1) cellular component (CC), (2) molecular function (MF), and (3) biological process (BP). KEGG is a reference for systematic interpretation of gene functions. An adjusted p-value below 0.05 was considered a significantly enriched gene.

### 2.7 Transcriptome analysis

Transcriptome differences between tumor tissues and normal tissues were analysed based on TCGA-BRCA bulk RNA-seq data using the R packages “limma,” “reshape2,” “ggplot2” and “ggpubr,” p-adjust <0.05 were considered statistically significant.

### 2.8 Diagnostic ROC evaluation

ROC curves based on TCGA-BRCA batch RNA-seq data were plotted against the core gene clusters using the R software packages “pROC”and “ggplot2” to assess their clinical diagnostic value.

### 2.9 CIBERSORT analysis

The relationship between core cluster genes and the levels of various types of immune cell infiltration in breast cancer tissues was analyzed based on TCGA-BRCA batch RNA-seq data using the R software packages “ggplot2,” “reshape2,” “ggpubr,” “dplyr,” “ggsci” and “RcolorBrewer,” An adjusted p-value below 0.05 was considered a significantly enriched gene. adjusted p-value below 0.05 was considered a significantly enriched gene.

### 2.10 Analysis of scRNA-seq dataset.

Single cell sequencing data were analyzed using the “Seurat” software package. The GSE180286 dataset was first subjected to quality control (QC), retaining cells with less than 20% of mitochondrial genes and genes expressed in at least 3 cells in the expression range of 200–7500. We then identified highly variable genes for subsequent analyses, setting the number of highly variable genes at 2000. We used the “Harmony” software package to remove batch effects from the sample data. We constructed cell clusters using the “FindClusters” and “FindNeighbors” functions and used the “t-SNE” method to Visualisation. Finally, we performed cell annotation using “SingleR” and corrected for marker genes using different cell types.

The “ssGSEA” software package was used to quantify the activity of specific sets of genes in each cell. To analyze the differentially expressed genes (DEGs) between the two groups, we used the “FindMarkers” feature of the Seurat package. Statistical significance of genes (DEGs) was calculated using the Wilcoxon Wilcoxon test (p.adj<0.05) and other parameters were set to default values.

### 2.11 Cell culture

MDA-MB-231, MCF-7, 4T1, and MCF-10A were purchased from the cell bank of the Chinese Academy of Science. under the condition of 37°C incubator containing 5% CO_2_, Dulbecco’s Modified Eagle Medium (DMEM, high glucose) (11965126, Thermo Fisher, America) containing 10% fetal bovine serum and 1% P/S was used to culture MDA-MB-231, MCF-7, and 4T1, MCF-10A was cultured using MCF-10A-specific medium (CM-0525, Pricella, China).

### 2.12 Preparation of drug

DHIE was purchased from GlpBio (GC38184, GlpBio, America), and 20 mg of DHIE lyophilised powder was dissolved in 306.4 μL DMSO to obtain a stock solution with a final concentration of 200 mM.

### 2.13 Cell counting kit-8 assay

In CCK-8 experiments, cells were evenly seeded in 96-well plates at a density of 1 × 10^3^ cells/well, and 5 replicate wells were set up, incubated at 37°C for 4 h waiting for the cells to attach to the wall and then changed, and 10ul of Cell Counting Kit-8 reagent was added to each well (GK10001,GlpBio, Montclair, Californian, United States of America), and the reaction was carried out under light-avoidance conditions for 30 min to 1 h, followed by detection of optical density values in the 450 nm range using an enzyme marker. The assay was repeated at 24 h intervals for a total of 5 days using this time point as a reference.

### 2.14 Flow cytometry analysis

The effect of DHIE on the cell cycle progression of breast cancer was detected using the Cell Cycle Staining Kit (CCS01, MULTI SCIENCES, China), and the cells were processed by flow cytometry. Data were processed and analyzed using Flow Jo software (version 10.0.7).

### 2.15 Molecular docking

In order to further verify whether DHIE can work in combination with the core gene cluster targets screened above, we first obtained the chemical structure of DHIE from PubChem database. Then we obtained the receptor domains of AURKA, AURKB, CCNA2, CCNE1, CCNE2, CDC7, CDC25A, CDC25B, CDK1, CHEK1, PLK1, PLK4, and TYMS from the PDB database, and modified the protein by deleting ligands and water molecules, and adding hydrogen bonds using Pymol. Pymol was used to modify the protein by deleting ligands, removing water molecules, and adding hydrogen bonds. Finally, AutoDockTools-1.5.7 was used to perform molecular docking analysis and obtain the corresponding binding sites and binding energies.

### 2.16 Western blot analysis

Non-denatured tissue cell lysate was formulated with protease inhibitor mixture and protein phosphatase inhibitor mixture in the ratio of 100:1:1 to prepare cell extracts. The supernatant containing cellular proteins was extracted and quantified using the BCA protein concentration assay kit (P0012, Beyotime, Shanghai, China), so as to prepare 1 μg/μL consisting of protein supernatant, 5×loading buffer (P0286, Beyotime, Shanghai, China) and high-pressure purified water to make up the upper sample, which was then placed in a 100°C heater and boiled for 10 min. PAGE polyacrylamide gels of corresponding concentrations were prepared using 6% or 10% PAGE gel rapid preparation kits (PG110, PG112, Epizyme, Shanghai, China), and the same amount of protein samples was added to complete electrophoretic separation. The membrane was then transferred to a polyvinylidene fluoride (PVDF) membrane or nitrocellulose (NC) membrane and closed with protein-free rapid closure solution (PS108P, Epizyme, Shanghai, China) for 15 min, and the membrane was incubated with primary antibody overnight at 4°C. The strips were washed three times with PBST buffer, each time at an interval of minutes. Subsequently, the membrane was incubated with secondary antibody coupled with HRP for 1 h at room temperature and then washed three more times. The intensity of the strips was visualized by a digital gel imaging system using chemiluminescent solutions. Grey scale values were analyzed using ImageJ software and all images were normalized. The following antibodies were used: PLK1 Polyclonal antibody (10305-1-AP, proteintech, America), Ki67 Polyclonal antibody (27309-1-AP, proteintech, America), p53 Polyclonal antibody (10442-1-AP, proteintech, America), CDK4 Polyclonal antibody (11026-1-AP, proteintech, America), GAPDH Polyclonal antibody (10494-1-AP, proteintech, America), biotin efficiently labelled goat anti-mouse IgG(H + L) (A0288, Beyotime, Shanghai, China), biotin efficiently labelled goat anti-rabbit IgG(H + L) (A0279, Beyotime, Shanghai, China).

### 2.17 Animal Assays

The mice were randomly divided into the following groups: the NC group, the DHIE treatment group, the DOX treatment group, and the combined treatment group. Each group contained five mice. To establish a subcutaneous hormonal tumor model in mice, 4T1 cells were injected subcutaneously into the right axilla of the mice. The digested 4T1 cells were resuspended in phosphate-buffered saline (PBS) to ensure a cell concentration of 1 × 10^7^ cells/mL, and then 1 × 10^6^ cells were injected into 4-week-old female BALB/c mice. After waiting for the mice to develop tumor load for 1 week, the drug was administered continuously for 2 weeks according to the following regimen: 67.2 mg of lyophilized powder of DHIE was dissolved in 240 μL of DMSO and diluted with 8100 μL of PBS, yielding a final concentration of DHIE at 8 mg/L. Similarly, 7 mg of DOX was dissolved in 70 μL of DMSO and diluted with 21,000 μL of PBS, yielding a final concentration of DOX at 8 mg/L. An additional 7 mg of DOX was dissolved in 70 μL of DMSO and diluted with 21,000 μL of PBS, resulting in a final concentration of DOX at 0.3 mg/L 28 μL of DMSO was added to 1 mL of PBS, yielding a concentration of 2.8%. In the NC group, 8 mL/kg of sterile PBS was injected. In the DHIE treatment group, 40 mg/kg of configured DHIE drug solution was injected. In the DOX group, 1.5 mg/kg of doxorubicin was injected. In the combined treatment group, 1.5 mg/kg of doxorubicin was injected. The combined dose of DHIE and DOX was injected. The drug was injected intraperitoneally every other day, and the tumor size and body weight of the mice were measured and recorded seven times. The mice were euthanized after 3 weeks, and gross photographs were taken to measure the tumor volume and weight.

### 2.18 H&E staining

Subcutaneous hormonal tumor tissues from mice were collected and fixed using 4% paraformaldehyde. The fixed and finished tissues were put into embedding box, dehydrated according to the gradient of ethanol concentration, then embedded using wax block, sectioned, prepared and dried. The tissue sections were first immersed in hematoxylin dye for 1 min and dipped and washed in pure water for 5 times, then immersed in eosin dye for 10 s and dipped and washed in pure water until colorless and removed, then the sections were deparaffinized and blocked. All procedures were carried out in accordance with the regulations of the Chinese Ethics Committee for Laboratory Animals and observed under a microscope.

### 2.19 Immunohistochemical staining

The sectioning procedure was the same as that for H&E staining. Immunohistochemical staining was performed as follows: first, a gradient dewaxing and rehydration was performed, and antigen repair was completed by placing the sections into an antigen repair solution boiled over low heat. Then 3% hydrogen peroxide solution was added to inactivate the endogenous peroxidase, and PBST was used to complete the washing process. 1% BSA was added and the sections were placed in a wet box at room temperature for 1 h to complete the sealing process. The primary antibody was incubated overnight at 4°C according to the instructions of the immunohistochemical assay kit (PK10006, proteintech, America). On the next day, the secondary antibody was incubated at room temperature for 1 h, followed by the dropwise addition of DAB chromogenic solution for staining, and after staining was completed, the staining was immersed in running water for 2 h, and hematoxylin re-staining, deparaffinisation and sealing were performed. The positive staining area and signal intensity of immunohistochemical staining images were obtained by using the IHC Toolbox plugin in ImageJ and the H-DAB mode was selected. The expression of the same index in different groups was analyzed and compared by integrating the area and depth of positive signal and calculating the mean optical density (MOD) in the whole field of view. All procedures were carried out in accordance with the regulations of the Chinese Ethics Committee for Laboratory Animals. Images were obtained using a microscope.

### 2.20 Statistical analysis

All experimental data were entered into Excel and statistically analyzed using Graphpad prism 8.0. Data were expressed as mean and standard deviation (mean + SEM) and compared using unpaired two-tailed Student's t-test and analysis of variance (ANOVA) with post hoc analysis if there were significant differences in ANOVA, p < 0.05 indicated that the differences were statistically significant, *p < 0.05, **p < 0.01, ***p < 0.001 ****p < 0.0001.

## 3 Results

### 3.1 Screening of active ingredients and targets

**FIGURE 1:**
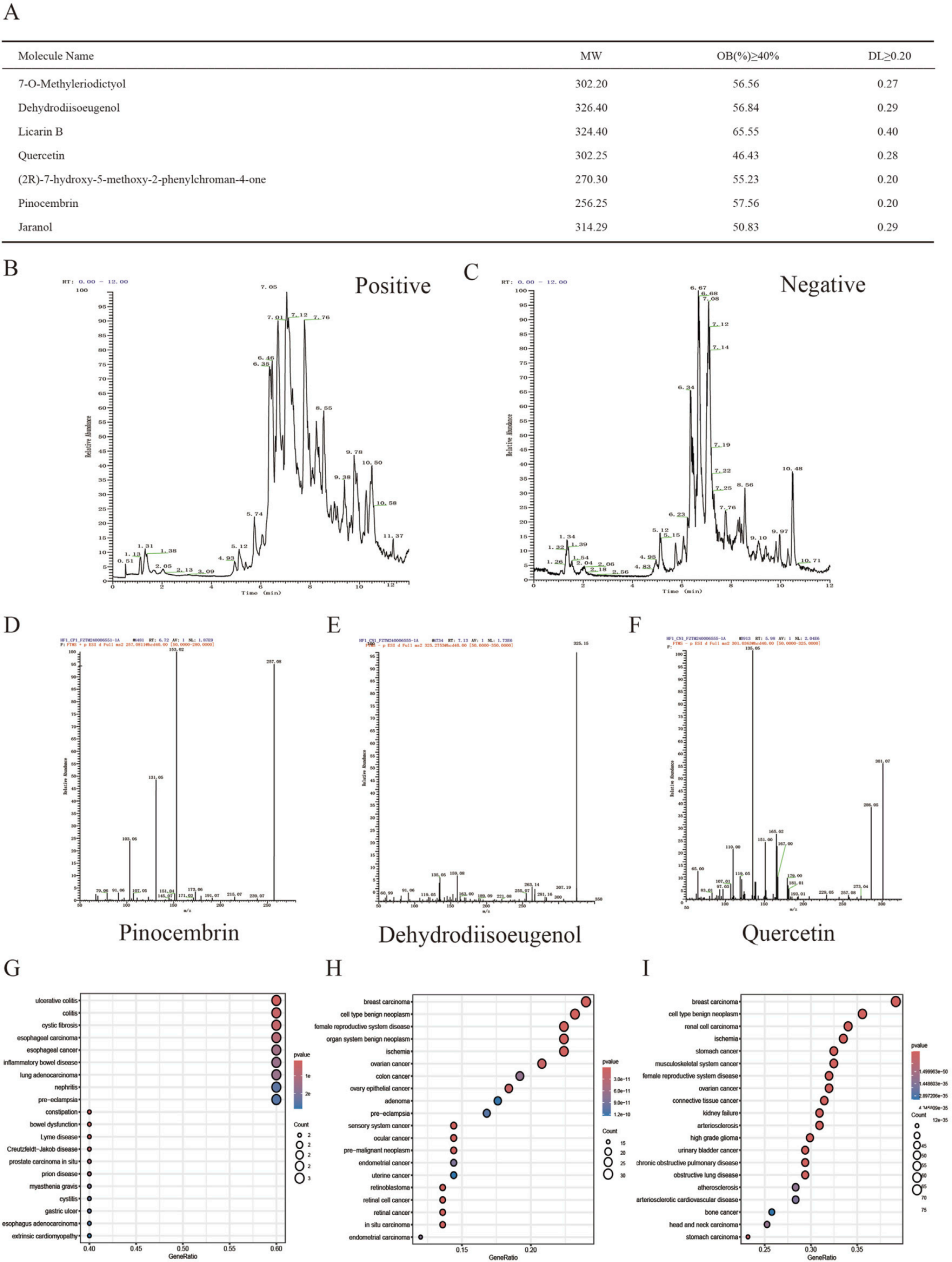
Qualitative and relative quantitative analysis of the components of AKH. **(A)** Seven compounds were screened in the TCMSP database using OB ≥ 40% and DL ≥ 0.20 as criteria. **(B, C)** LCMS analysis of panoramic chromatograms of AKH in positive and negative ion modes, respectively. **(D, E, F)** Three compounds further screened by combining TCMSP database with LCMS analysis. **(G, H, I)** Collection of the targets of the above three compounds for DO enrichment analysis.

Initially, the TCMSP database was searched for AKH, revealing 71 bioactive components. Then, after ADME screening based on the criteria of OB ≥ 40% and DL ≥ 0.20, seven candidate compounds were further identified as the active bioactive components of AKH (Figure1A). LC-MS was used to identify the bioactive components of AKH and their relative contents and to show the total ion chromatograms of all its components in positive and negative ion collection mode (Figures 1B, C). On the basis of the bioactive components screened in the TCMSP database above combined with the presence of components identified by LC-MS, three bioactive components, pinocembrin, dehydrodiisoeugenol and quercetin, were further screened (Figures 1D–F). The targets of the above three components were collected and analyzed for DO enrichment in several databases such as TCMSP, CTD, SwissTargetPrediction and PharmMapper, which showed that there was no breast cancer among the diseases enriched for the target of pinocembrin. On the other hand, dehydrodiisoeugenol and quercetin showed the highest correlation with breast cancer, and the relative amount of dehydrodiisoeugenol (RT = 7.13 min) was significantly higher than that of quercetin (RT = 5.98 min) in the negative ion collection mode. Therefore, DHIE was chosen to study its mechanism of action on breast cancer in depth (Figures 1G–I).

**FIGURE 2:**
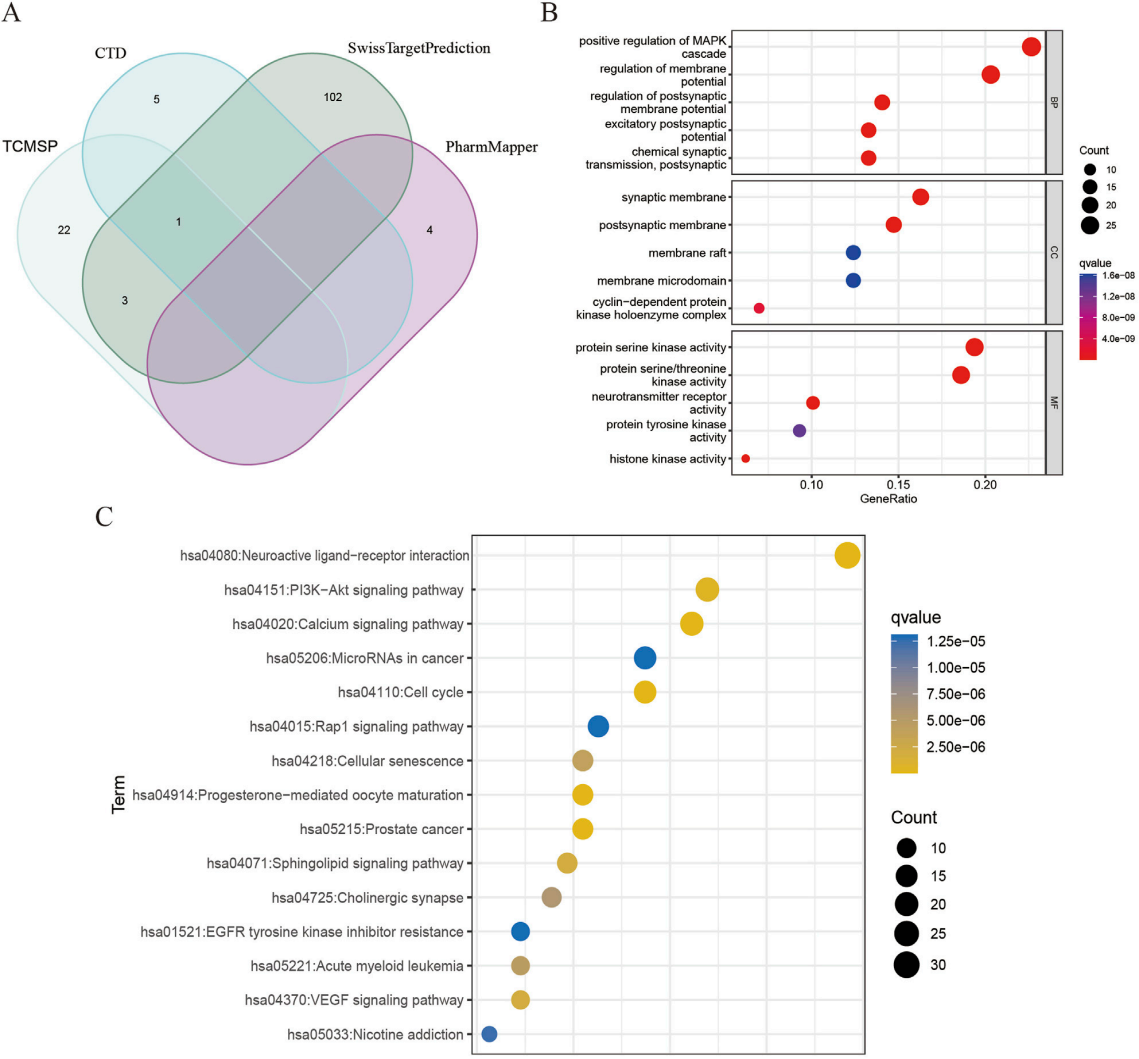
Analysis of targets and enrichment related to DHIE. **(A)** A total of 137 fusion targets for DHIE were identified from the TCMSP, CTD, SwissTargetPrediction, and PharmMapper databases. **(B)** Gene Ontology analysis categorizes the drug targets into three distinct components: cellular component (CC), molecular function (MF), and biological process (BP). **(C)** KEGG pathway enrichment analysis was conducted on the drug targets.

A total of 137 targets of DHIE were integrated in TCMSP, CTD, SwissTargetPrediction and PharmMapper databases (Figure 2A). GO enrichment analysis of the above targets showed that DHIE was mainly involved in the positive regulation of MAPK cascade and regulation of membrane potential, and acted on synaptic membrane and cyclin-dependent protein. and cyclin-dependent protein kinase holoenzyme complex, and influences protein serine/threonine kinase activity and neurotransmitter receptor molecular function (Figure 2B). KEGG enrichment analysis was performed to collect drug targets, and the results showed that DHIE mainly regulated the Neuroactive ligand-receptor interaction,PI3K-Akt signaling pathway and Cell cycle and other related signaling pathways (Figure 2C).

### 3.2 Combined WGCNA screening of differentially expressed genes as breast cancer targets

**FIGURE 3:**
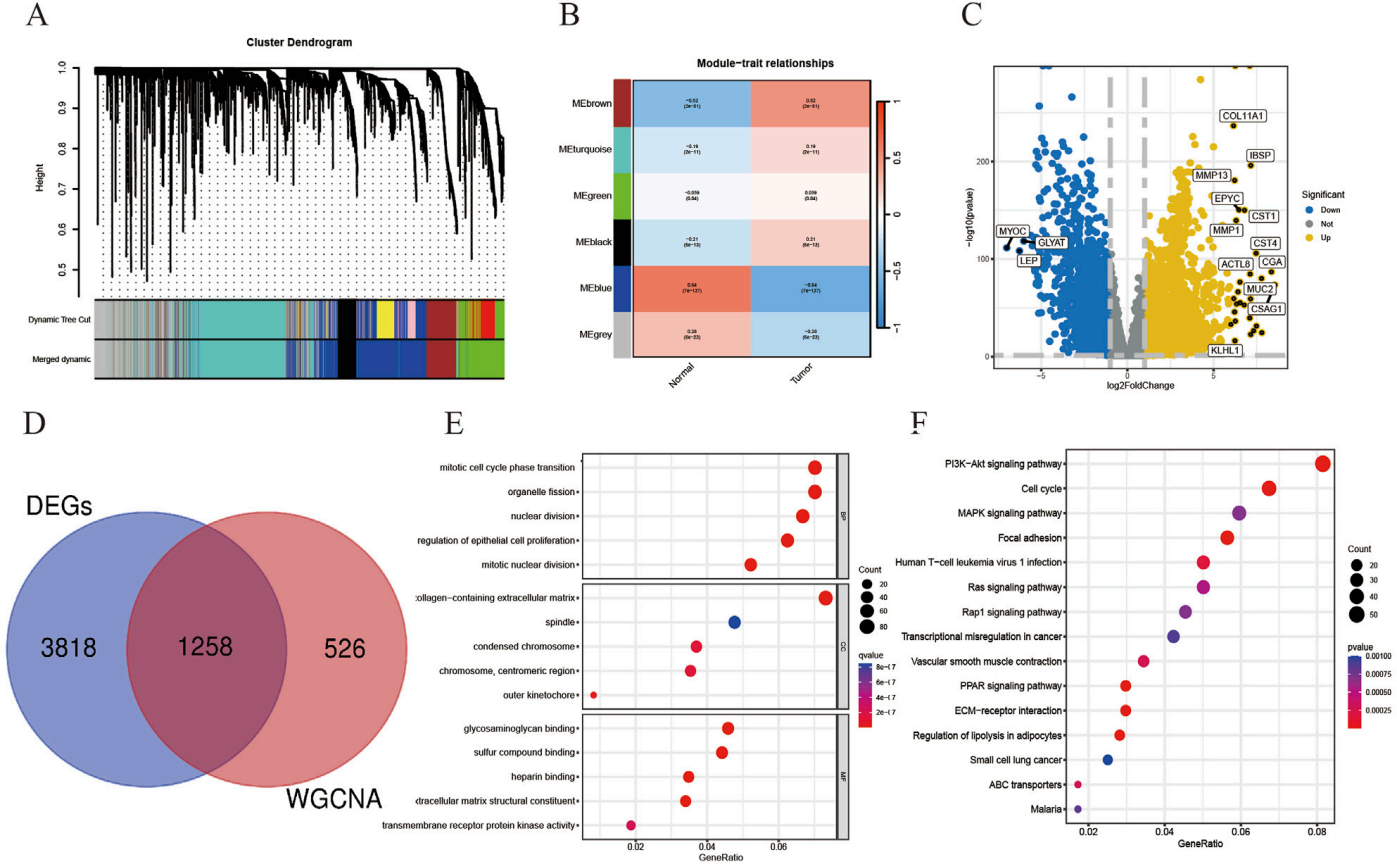
On the basis of breast cancer co-expression module genes, relevant differentially expressed genes were screened as breast cancer disease targets and related enrichment analysis was performed. **(A)** Gene hierarchical-tree clustering diagram. The figure shows the distribution of expression levels of different gene groups, the Irrelevance between genes, the lower the branch, the smaller the Irrelevance of genes within the branch, that is, the stronger the correlation. **(B)** Heat map showing the relationship between modules and tumor characteristics. The values in the small cells of the figure represent the two calculated correlation values cor coefficients between each feature and the eigenvalues of each module and the corresponding statistically significant p-values. The color corresponds to the magnitude of the correlation, The darker the red, the more positive the correlation, The darker the blue, the more negative the correlation. **(C)** Volcano plot showing DEGs in the TCGA-BRCA dataset. **(D)** There were 1258 targets in the intersection of cluster module genes and DEGs with strong WGCNA correlation. **(E)** Gene Ontology analysis of the above-mentioned intersection disease targets was performed in three parts: cellular component (CC), molecular function (MF), and biological process (BP). **(F)** KEGG pathway enrichment analysis of disease targets.

We downloaded TCGA-BRCA from the TCGA database. The breast cancer data in TCGA-BRCA was used as the experimental dataset for this study, consisting of 1081 breast cancer samples and 99 paraneoplastic normal samples. The co-expression trends of all genes in this dataset were analyzed using WGCNA, and several module genes whose traits were associated with tumor or normal were clustered and stratified, among which the genes in brown module and blue module had obvious upregulation or downregulation trends and more significant correlations, so genes in these two modules were selected for the subsequent study (Figures 3A, B). Samples from the TCGA-BRCA dataset were used for the analysis of differentially expressed genes, and marker volcano maps were drawn (Figure 3C). The genes from the combined brown module and blue module were intersected with the differentially expressed genes to obtain 1258 genes as a more precise cluster of breast cancer targets for subsequent analysis (Figure 3D). The above breast cancer targets were collected for GO and KEGG enrichment analysis, and the results showed that the disease targets were mainly involved in biological processes such as mitotic cell cycle phase transition and organelle fission, acted on cellular components such as spindle and condensed chromosome, affected glycosaminoglycan binding and sulfur compound binding, as well as regulating PI3K-Akt signaling pathway, Cell cycle and MAPK signaling pathway (Figures 3E, F).

### 3.3 Network construction and enrichment analysis

**FIGURE 4:**
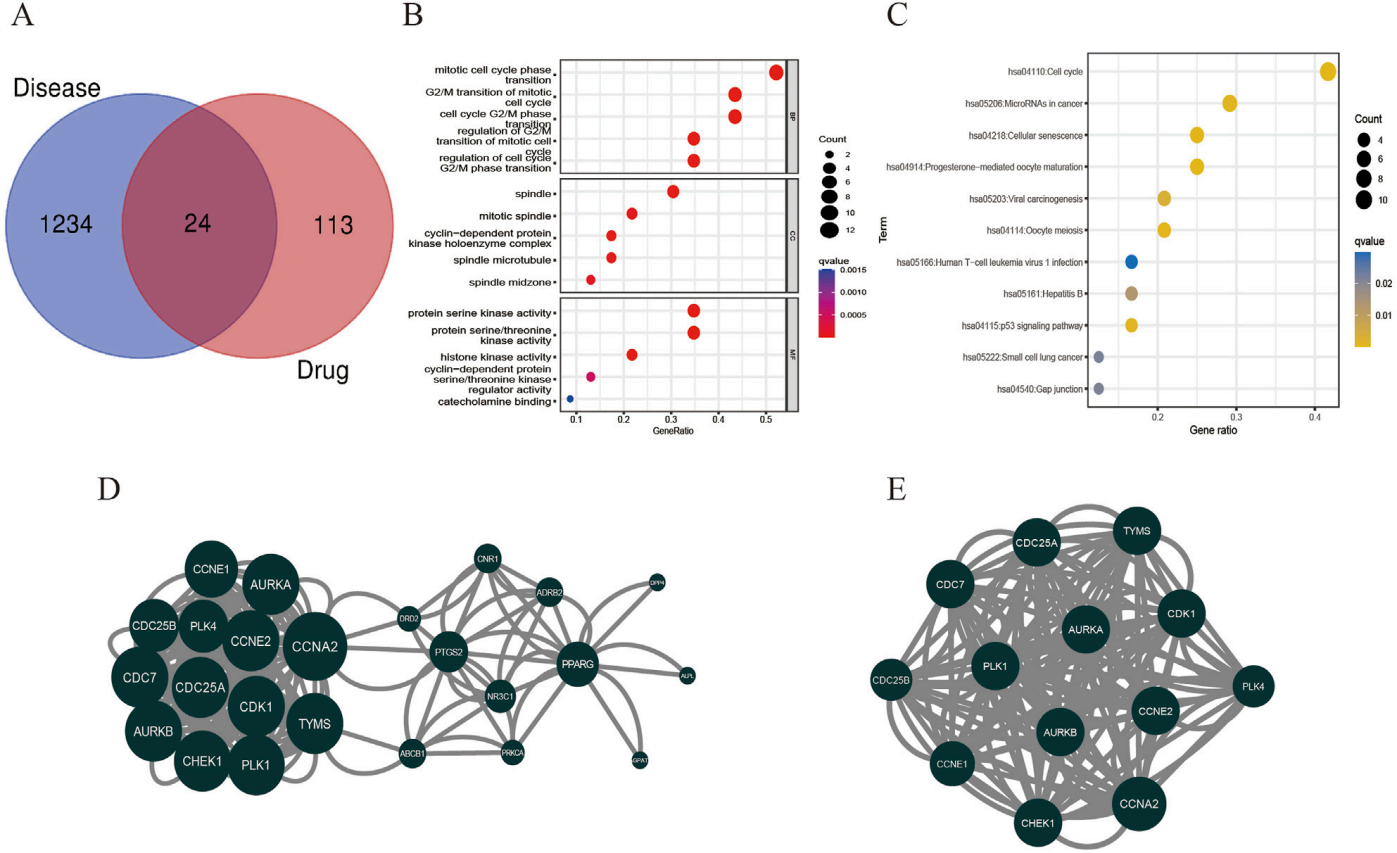
Network pharmacology analysis showed that DHIE may act by regulating cell cycle-related pathways in breast cancer cells. **(A)** Intersection and co-target of DHIE and breast cancer. **(B)** The intersection and common targets were selected for Gene Ontology analysis, which was divided into three parts: cellular component (CC), molecular function (MF), and biological process (BP). **(C)** KEGG pathway enrichment analysis of common targets. **(D)** The protein-protein interaction network (PPI) of co-targets was constructed by Cytoscape. **(E)** The core cluster gene network in PPI was obtained based on MCODE algorithm.

A network pharmacology approach was used to take the intersection of DHIE’s targets with breast cancer to obtain 24 co-targets (Figure 4A). The co-targets were analyzed by GO and KEGG enrichment, and the results showed that the main biological processes of DHIE affecting breast cancer were mitotic cell cycle phase transition, acting on cellular components such as spindle, regulating molecular functions such as protein serine kinase activity and other molecular functions, regulate cell cycle and p53 signaling pathway and other signaling pathways (Figures 4B, C). It can be seen that the effects of DHIE on breast cancer are mainly focused on the cell cycle, thus affecting the proliferation function of cancer cells. The 24 co-targets were constructed into a protein-protein interaction network (PPI) using Cytoscape 3.9.1, and a core subgroup of 13 genes was further obtained by combining with MCODE clustering analysis. (Figures 4D, E).

### 3.4 RNA sequencing combined with diagnostic ROC, immune infiltration and single cell sequencing analysis

**FIGURE 5:**
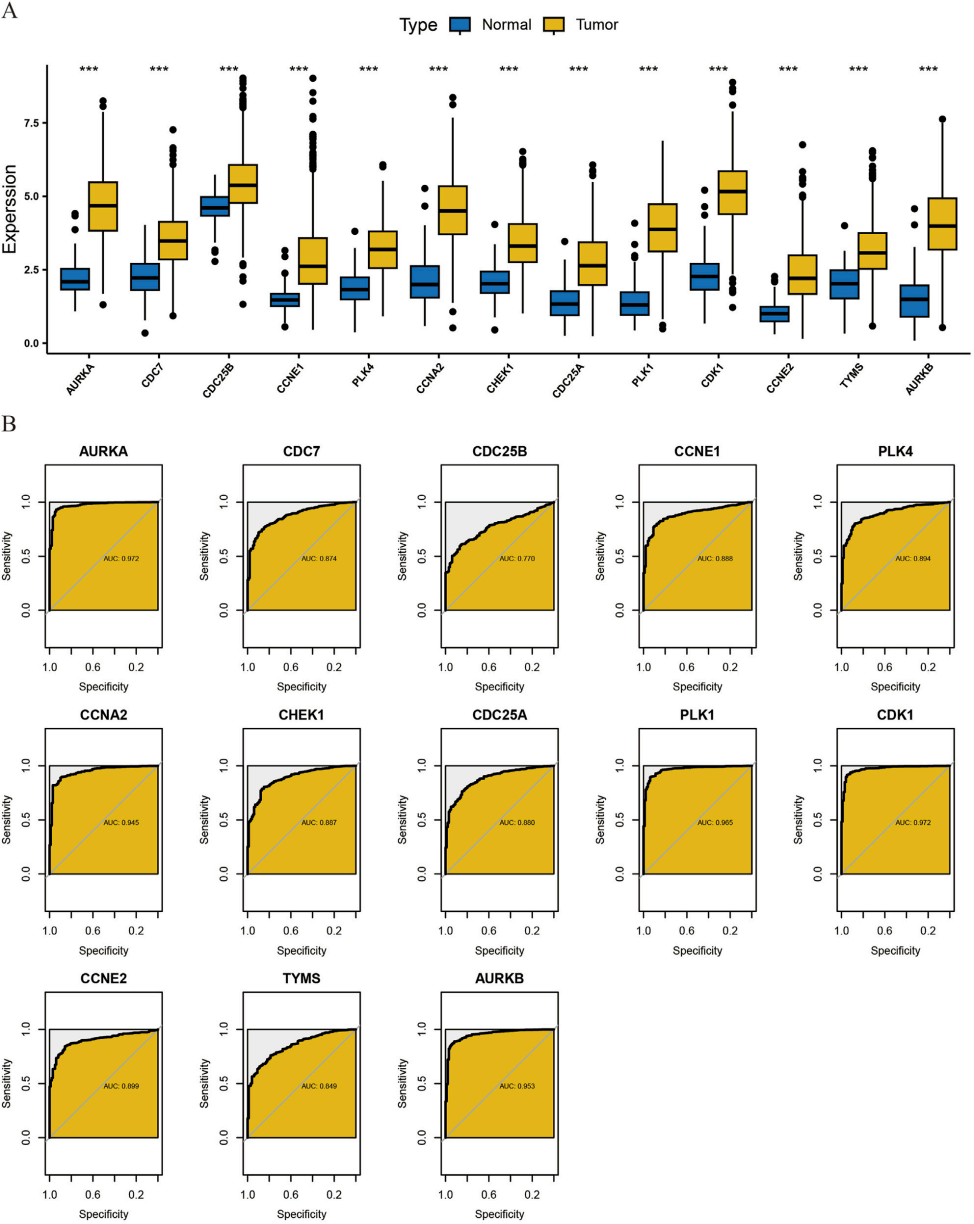
Transcriptome differential Analysis of core cluster genes and diagnostic ROC Evaluation. **(A)** To analyze the differences in the transcriptome level of core cluster genes obtained by MCODE algorithm between normal tissues and tumor tissues. **(B)** diagnostic ROC evaluation of core cluster genes.

We obtained relevant Bulk RNA-seq data based on 1081 breast cancer samples and 99 normal paracancer tissue samples in TCGA-BCRA. Based on the core gene clusters obtained from MCODE clustering analysis, we performed transcriptome difference analysis and found that all of the above core gene clusters were highly expressed in tumors and lowly expressed in normal tissues, suggesting that the gene clusters might be positively correlated with the tumor progression trend (Figure 5A).

Secondly, we found that this gene cluster has high clinical diagnostic value for breast cancer by diagnostic ROC evaluation analysis. The AUC of CDC7, CDC25B, CCNE1, PLK4, CHEK1, CDC25A, CCNE2, TYMS was between 0.7 and 0.9, which had moderate diagnostic accuracy. The AUC of AURKA, CCNA2, PLK1, CDK1, AURKB was above 0.9, which had high diagnostic accuracy (Figure 5B).

**FIGURE 6:**
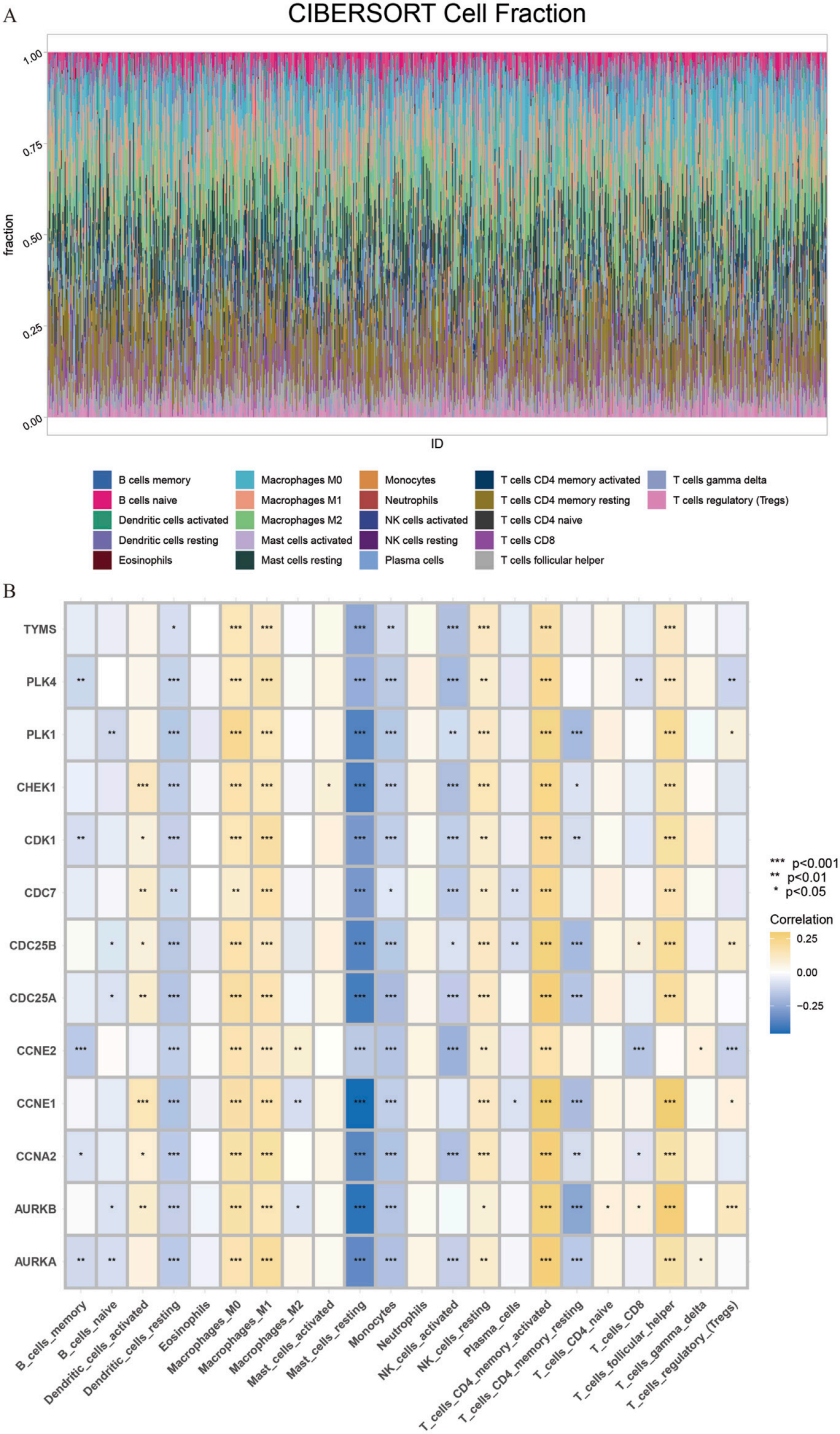
CIBERSORT immune Infiltration Analysis of core cluster genes. **(A)** The proportion of immune cell infiltration in 1081 breast cancer samples and 99 adjacent normal tissue samples obtained from the TCGA database was analyzed. **(B)** CIBERSORT immune infiltration analysis of core cluster genes on the regulation of different proportions of immune cells in breast cancer tissues, the darker the yellow, the greater the proportion of upregulated, The darker the blue, the greater the proportion of downregulation.

Subsequently, we performed immune cell infiltration ratio analysis based on the sample data to predict the relative abundance of different immune cell types in each sample (Figure 6A). In breast cancer samples characterized by cluster clusters consisting of 13 core genes, the infiltration of M0 macrophages, M1 macrophages and CD4^+^ memory T cells was significantly upregulated, whereas the infiltration of monocytes, resting dendritic cells and resting mast cells was significantly downregulated, which revealed a correlation between the core cluster genes and the immune microenvironment of breast cancer, the DHIE may act by simultaneously affecting the proportion of infiltration or function of some types of immune cells in the tumor microenvironment (Figure 6B).

**FIGURE 7:**
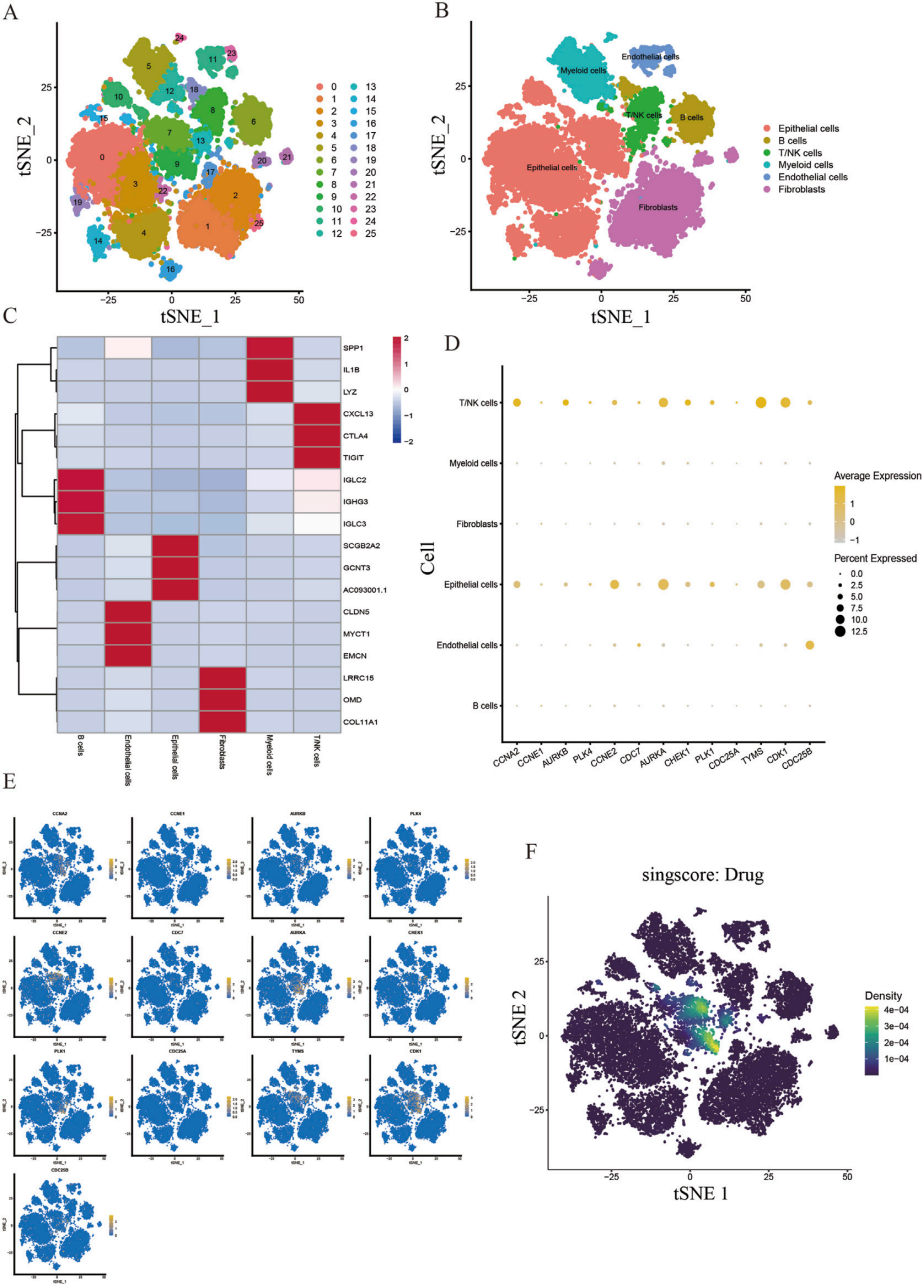
Single cell sequencing analysis of the core cluster gene action sites of DHIE regulating breast cancer. **(A)** T-distributed random neighborhood embedding map (tSNE) of 25 cell clusters. **(B)** Six cell types were identified by marker genes. **(C)** Top three marker genes for the six cell types. **(D)** expression levels of core cluster genes in six breast cancer cell types. **(E)** Cell-annotated distribution map of core cluster genes. **(F)** Cell distribution map of AUCell functional scores of drug targets.

We obtained scRNA-seq from the GSE180286 dataset and used the “Harmony” package to eliminate batch effects. Next, we performed principal component analysis (PCA) and t-distribution random neighbor embedding (t-SNE) on the top 2000 variant genes to reduce dimensionality. Cells were clustered into 25 clusters with a resolution of 1.0 (Figure 7A). Six clusters were distinguished using marker genes for different cell types, including epithelial cells, T/NK cells, B cells, myeloid cells, endothelial cells, and fibroblasts (Figure 7B). The heatmap shows the top three marker genes for each cell cluster (Figure 7C). We used the “AUCell” software package to analyze the enrichment distribution of the core gene clusters on each cell type, and labeled and visualized the expression levels of each gene in different cell types (Figures 7D, E). Combining the cellular enrichment distributions of the core gene clusters, it was found that the drug targets were mainly concentrated in epithelial cells in breast cancer tissues (Figure 7F).

### 3.5 DHIE inhibits proliferation of breast cancer cells by blocking their G0/G1 phase

**FIGURE 8:**
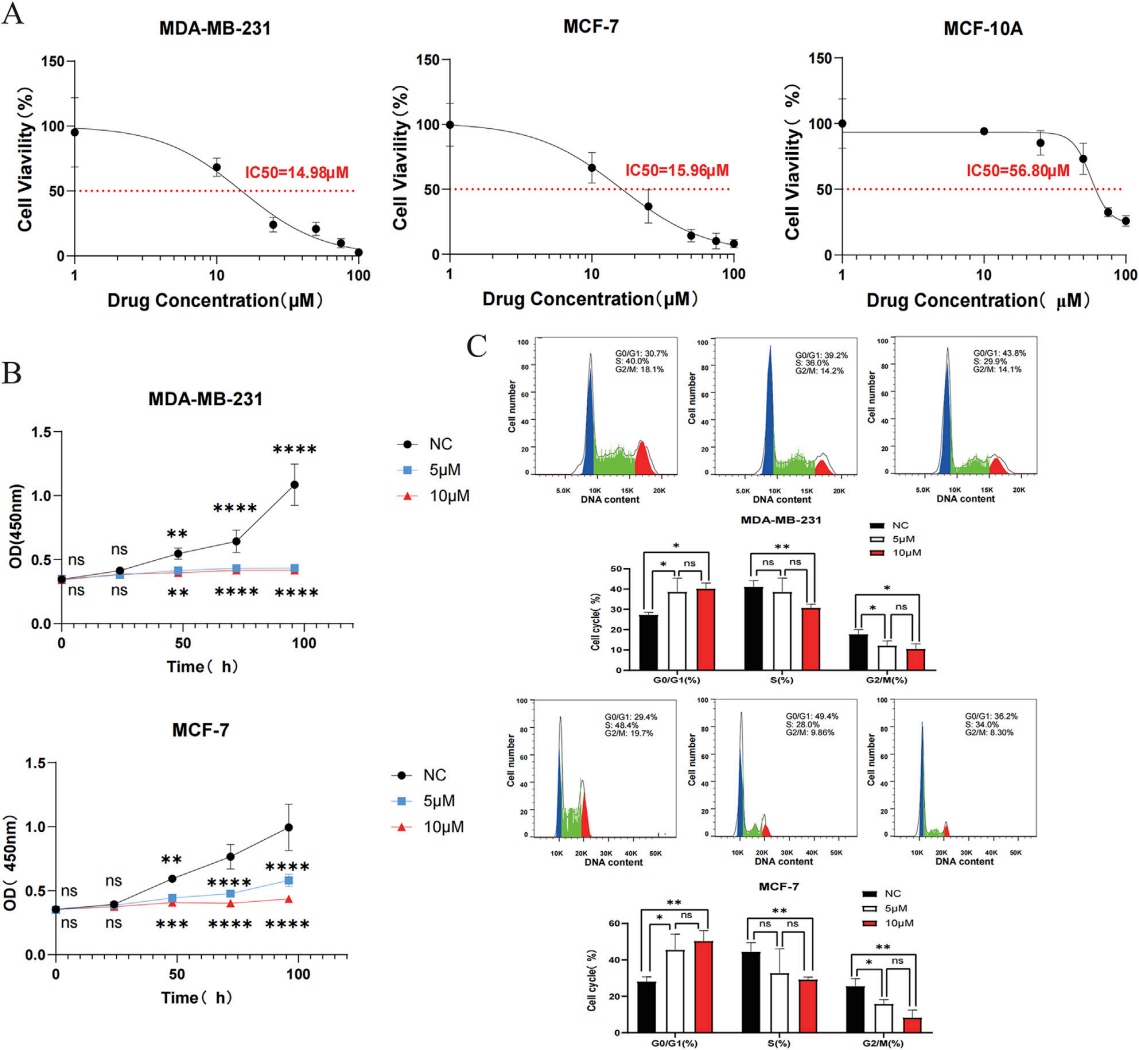
DHIE can regulate cell cycle to breast cancer cell proliferation inhibition function into full play. **(A)** IC50 of DHIE on MDA-MB-231, MCF-7 and MCF-10A cells was determined by CCK-8 assay. **(B)** CCK-8 assay showed that DHIE effectively inhibited the proliferation of MDA-MB-231 and MCF-7 cells. **(C)** Flow cytometry showed that DHIE could block DNA replication of MDA-MB-231 and MCF-7 cells at G0/G1 phase and reduce the percentage of cells entering G2/M phase. The cv values of all parameters were less than 10%. Data are expressed as the mean ± SD. *p < 0.05, **p < 0.01, ***p < 0.001 and ****p < 0.0001 vs. NC.

Firstly, the IC50 of two breast cancer cell lines MDA-MB-231 and MCF-7 and a normal breast epithelial cell line MCF-10A were 14.98 μM, 15.96 μM and 56.80 μM, respectively, by CCK-8 assay. The results showed that DHIE effectively killed breast cancer cells within a certain concentration range, but had no killing effect on normal epithelial cells, which ensured the safety of DHIE as a drug monomer on normal human tissues (Figure 8A). Secondly, we carried out CCK-8 cell proliferation experiment, divided into nc group, low concentration treatment group and high concentration treatment group, and found that DHIE treatment for 48 h could effectively inhibit the proliferation of MDA-MB-231 and MCF-7 (Figure 8B). Flow cytometry showed that the cell cycle of MDA-MB-231 and MCF-7 cells was arrested at G0/G1 phase after serum starvation for 24 h and induction with DHIE for 24 h. It effectively reduced the percentage of cells entering the G2/M phase and thus inhibited tumor proliferation (Figure 8C).

### 3.6 DHIE modulates the PLK1-p53 axis and thereby affects the cell cycle in breast cancer

**FIGURE 9:**
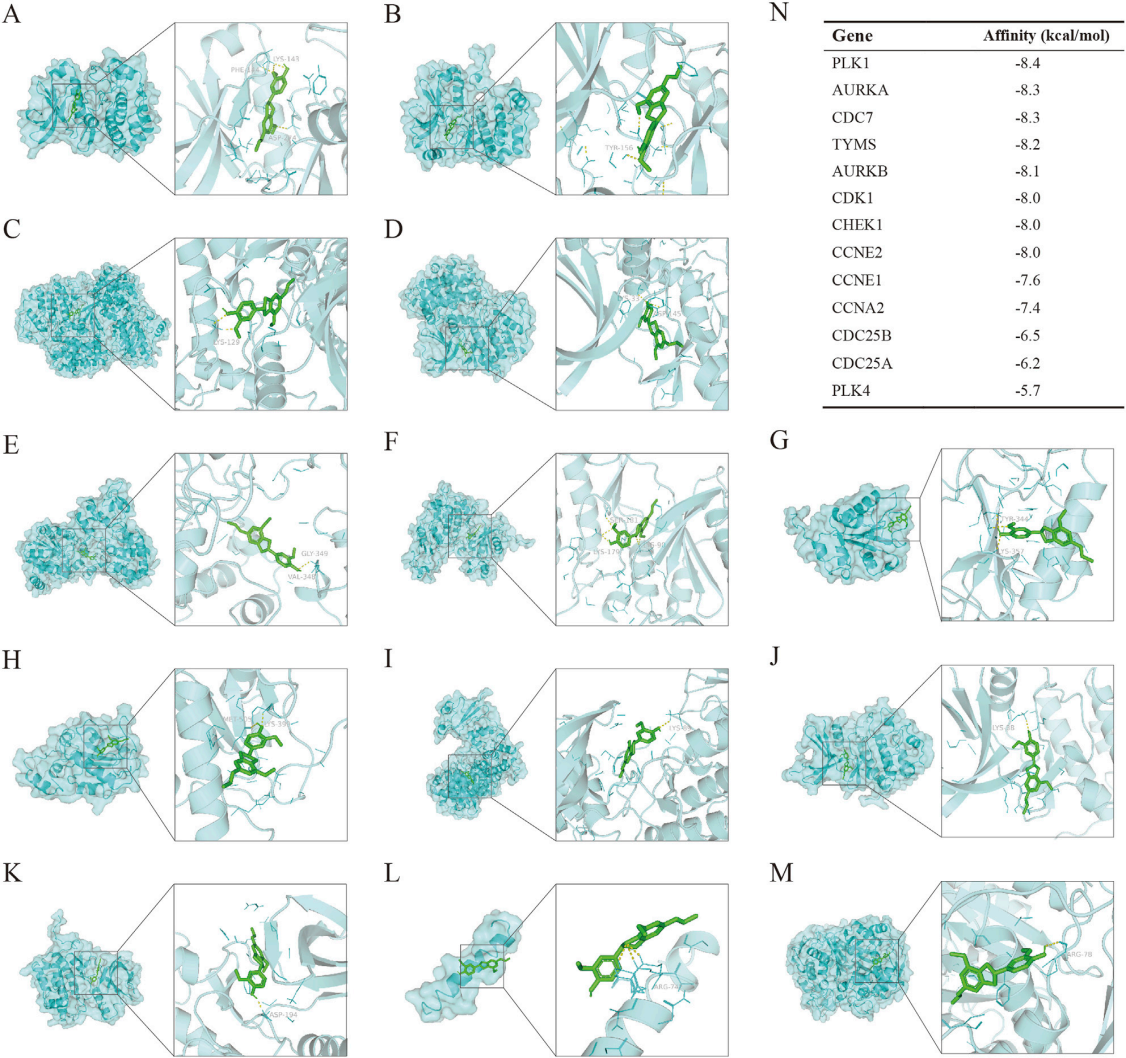
Molecular docking to measure the affinity of DHIE to the protein receptors expressed by core cluster genes. **(A–M)** molecular docking showed the docking conformation of ligand and receptor. DHIE could stably bind to the protein receptors expressed by 13 core cluster genes with binding energies less than −5.0 kcal/mol. Among them, the binding to PLK1 was the most stable, and the binding energy was −8.4 kcal/mol. **(N)** Binding energy of DHIE to the protein receptors expressed by each gene.

To verify the binding of DHIE to the protein receptors transcribed from the core cluster genes, we obtained the proteins transcribed and translated from the 13 genes of this cluster from the PDB database according to the accuracy of the resolution, and also set up the active pockets for docking using AutoDockTools-1.5.7 after completing a series of modifications such as dehydrogenation. The results showed that the binding energies of DHIE docked with the above proteins were all less than -5 kcal/mol, indicating that the drugs were all stably bound to them (Figures 9A–M). The binding energy of DHIE to PLK1 was the lowest at −8.4 kcal/mol, indicating that this binding conformation was the most stable compared to the other protein binding conformations (Figure 9N).

**FIGURE 10:**
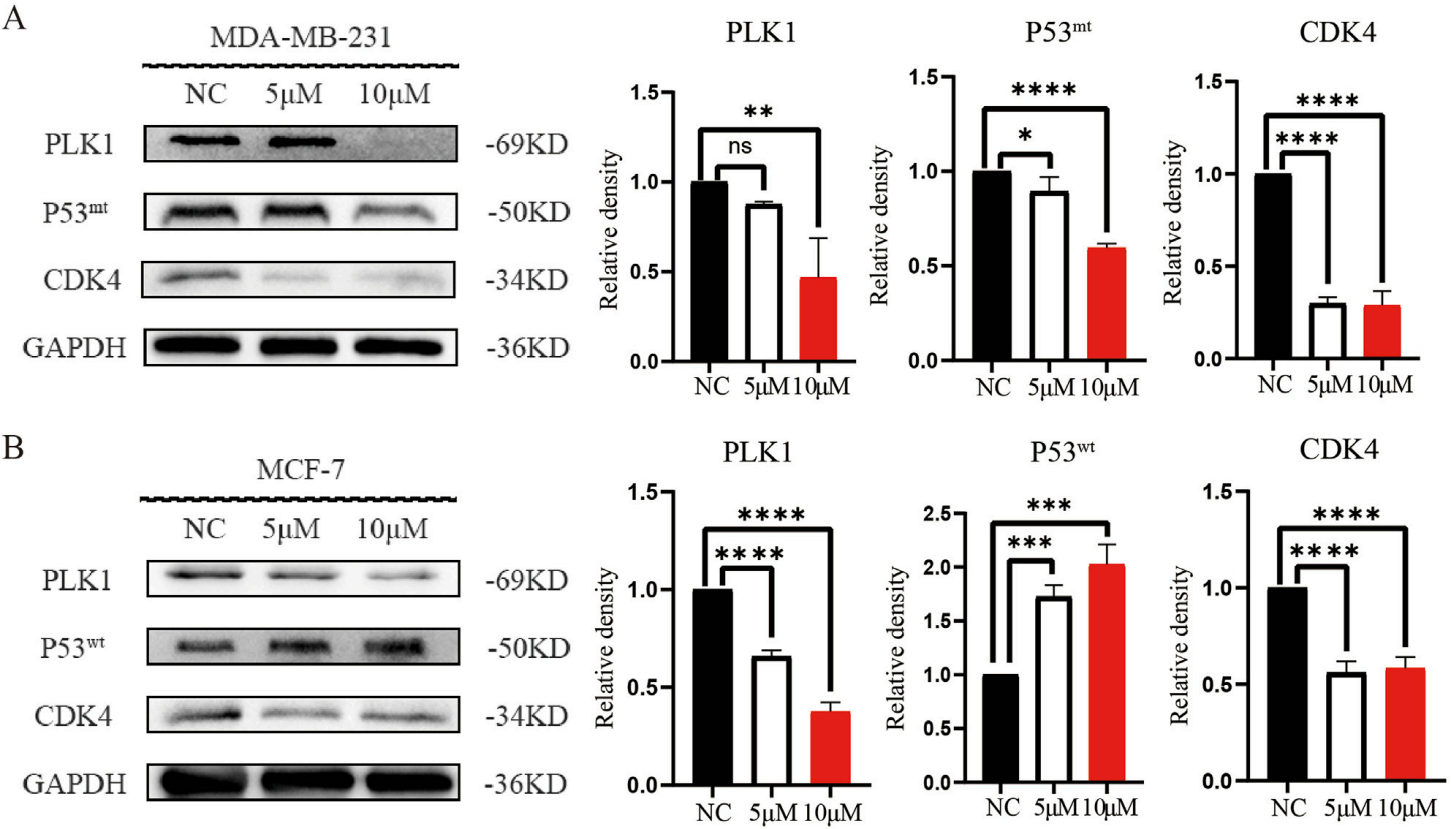
DHIE through regulating PLK1 - p53 signal shaft cause breast cancer cell cycle arrest. **(A)** Western blot analysis showed that DHIE inhibited the expression of PLK1 and caused the downregulation of Mtp53, which resulted in the decrease of CDK4 expression in MDA-MB-231. **(B)** For MCF-7, DHIE inhibited PLK1 expression and caused an upregulation of Wtp53 expression, which resulted in a decrease in CDK4 expression. All the above were dose dependent. Data are expressed as the mean ± SD. *p < 0.05, **p < 0.01, ***p < 0.001 and ****p < 0.0001 vs. NC.

Based on the KEGG pathway enrichment analysis and in conjunction with the relevant literature, we hypothesized that DHIE may regulate p53 by inhibiting PLK1, thereby blocking the cell cycle of breast cancer. Therefore, we examined the relevant molecular indexes in the NC, 5 μM and 10 μM treatment groups by Western blot and found that DHIE inhibited the expression of PLK1 and reduced the expression of mutant p53 in MDA-MB-231, which ultimately caused a decrease in the expression of the cell cycle marker CDK4 (Figure 10A). For MCF-7, DHIE was also able to inhibit PLK1 expression, but the difference was that p53 within MCF-7 was wild-type. And our experimental results showed that DHIE was able to upregulate the expression of wild-type p53 in MCF-7, thus exerting an oncogenic function and causing downregulation of CDK4 expression (Figure 10B). Based on the above results, we believe that DHIE may inhibit PLK1 to downregulate p53^mt^ or upregulate p53^wt^, and ultimately cause breast cancer cell cycle arrest and play an oncogenic role.

### 3.7 DHIE improves survival and inhibits tumor progression in a mouse model of breast cancer

**FIGURE 11:**
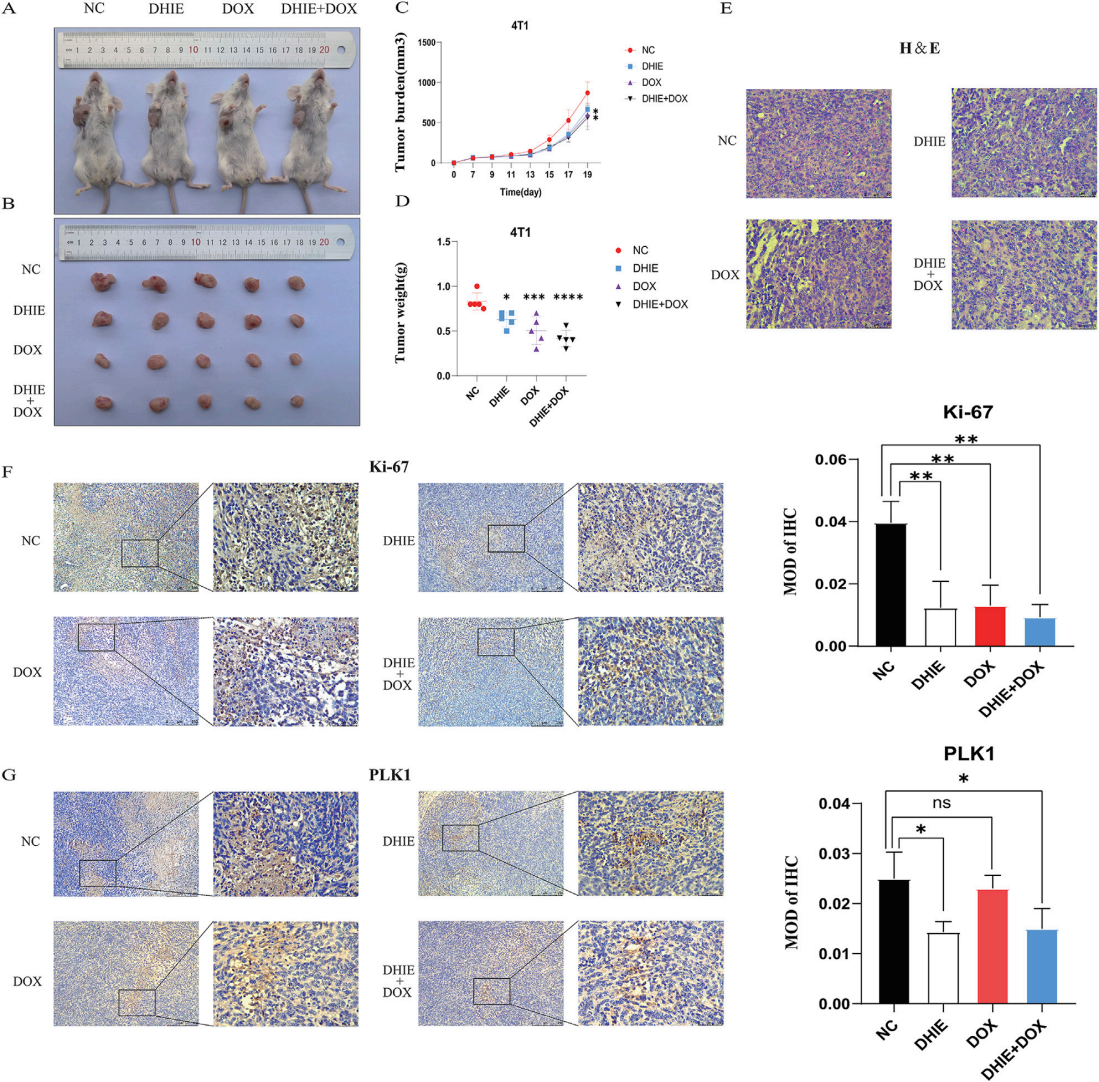
*In vivo* experiments showed that DHIE could inhibit the development of subcutaneous tumor bearing mice, downregulate the expression level of PLK1 in the tumor bearing mice, and improve the survival of mice. **(A, B)** Representative images of the gross appearance of mice and tumors were taken after the mice were sacrificed. **(C, D)** Comparison of the changes in tumor volume and the final weight of the tumor-bearing mice showed that compared with the NC group, the DHIE treatment group, DOX treatment group and combined treatment group could effectively improve the survival of the mice. **(E)** The morphology of tumor cells in tumor-bearing sections of each group was observed under high power microscope. **(F, G)** Immunohistochemical staining showed that DHIE, DOX and combined treatment all reduced the expression of Ki-67 in the tumor bearing cells and inhibited tumor proliferation. DHIE treatment significantly reduced the expression level of PLK1 in the tumor-bearing mice. N = 5 for each group. Data are expressed as the mean ± SD. *p < 0.05,***p < 0.001 and ****p < 0.0001 vs. NC.

In order to investigate the anti-tumor effects of DHIE *in vivo* when used alone or in combination with clinical chemotherapeutic agents, we firstly used 4T1 to construct a subcutaneous loaded tumor model in 4-week-old BALB/c mice, and then continuously injected mice intraperitoneally according to the drug administration regimen, and then the mice were executed after 3 weeks, and the tumors were taken to complete fixation, sectioning and staining. The general results showed that compared with the NC group, the DHIE treatment group, the DOX treatment group and the combined treatment group were able to significantly improve the survival and prognosis status of the mice and reduce the tumor load of the mice (Figures 11A–D), and the effect of the combined treatment group was the most obvious, which indicated that the DHIE combined with the DOX treatment strategy was able to improve the degree of infiltration of the cancer cells in the tumor tissues to a certain extent (Figure 11E), and reduce the side effects of chemotherapy to a certain extent.

In addition, immunohistochemical staining analysis showed that the DHIE treatment group was able to downregulate the degree of Ki-67 infiltration in subcutaneous hormonal tumors and inhibit the proliferation of cancer cells in tumor tissues (Figure 11F). At the same time, compared with the DOX treatment group, the DHIE treatment group was able to significantly reduce the expression level of PLK1 in subcutaneous homozygous tumors, indicating that DHIE may inhibit proliferation by down-regulating the expression of PLK1 in tumors (Figure e11G).

## 4 Discussion

Breast cancer, a disease with high incidence and mortality among women, sees significant improvements in the 5-year survival rate due to the current strategy of neoadjuvant chemotherapy followed by surgery and adjuvant chemotherapy. Nonetheless, approximately 50% of patients with intermediate and advanced stages still confront the serious issue of chemotherapy resistance (An et al., 2021). As natural compounds, Chinese herbs have a wide range of pharmacological activities. In recent years, many active ingredients of Chinese herbs have been used in clinical adjuvant treatment of tumors, which suggests that exploring the mechanism of action of active ingredients of natural medicines on breast cancer can provide new clinical solutions to the dilemma of chemotherapy resistance.

Our review of the literature revealed that relevant studies have shown that AKH and some of its active ingredients can inhibit the progression of breast cancer (Zhang et al., 2020). However, the current study lacks relevant evidence to show which active components in AKH mainly exert their effects against breast cancer, and through which active components AKH mainly functions as a breast cancer inhibitor in human beings and its mechanism of action is not clear. In this study, we first obtained the active components with high oral utilization and bioavailability in AKH as potential drug monomers based on the TCMSP database. Subsequently, we further screened pinocembrin, dehydrodiisoeugenol and quercetin by qualitative as well as relative quantification of the active components of AKH by LC-MS. The targets of these three compounds were collected for disease enrichment analysis, and the results showed that the disease with the highest correlation rank of DHIE was breast cancer, with the highest relative quantification under the same collection mode. Therefore, it is reasonable to assume that DHIE is the main active ingredient in AKH that exerts anti-breast cancer effects. Based on various bioinformatics analyses such as network pharmacology, transcriptome difference analysis, single-cell sequencing analysis, and molecular docking, we initially obtained 13 core gene clusters in which DHIE affects breast cancer, and screened PLK1 as the target with the highest relevance, which may exert an inhibitory effect on cancer by blocking the cell cycle. Then, we carried out *in vivo* and *ex vivo* experiments to validate the results, and further found that DHIE could inhibit PLK1 in different subtypes of breast cancer, and then regulate the expression of p53 (specifically, downregulate the expression of p53 in triple-negative breast cancer mutant type or upregulate the expression of p53 in wild-type of HER2-overexpressing breast cancer), which resulted in the outcome of the cell-cycle blockade in breast cancer.

The cell cycle is a series of phases that cells go through during growth and division, and is usually categorized into G1, S, G2 and M phases. The regulation of the normal cell cycle is essential for maintaining cell growth and development. However, in malignant tumors such as breast cancer, alterations in certain cell cycle proteins or kinases allow the cell cycle regulatory mechanisms to be often disrupted, leading to excessive cell proliferation and tumor formation (Butt et al., 2008). Due to the critical role of the cell cycle in breast cancer, many studies are exploring therapeutic strategies that target the cell cycle. For example, mid-G1 is controlled by CDK4 and CDK6, two serine/threonine kinases whose catalytic activity is regulated by D-type cell cycle proteins (D1, D2, and D3). When CDK4/6 is inhibited, it restricts tumor cells from entering the next stage of mitosis (Piezzo et al., 2020), increasing evidence suggests that dysregulation of long-chain non-coding RNA (lncRNAs) is closely related to the occurrence and progression of breast cancer. lncRNA taurine upregulated gene 1 (TUG1), also known as lncRNA00080, has a low expression level in breast cancer tissues and cells at a low level in breast cancer tissues and cells. Silencing TUG1 promotes BC cell cycle progression through the upregulation of cell cycle protein D1 and cell cycle protein-dependent kinase 4 (CDK4) (Iliaki et al., 2021), natural active compounds combined with first-line chemotherapeutic agents are increasingly being studied. Quercetin, a popular flavonoid with low adverse effects and antitumor properties, significantly enhanced the cell cycle arrest and apoptosis-inducing effects of adriamycin on breast cancer T47D cells when combined with adriamycin (Azizi et al., 2022). Therefore, the development of inhibitors against CDK4/6, the development of drug candidates targeting lncRNAs and the screening of natural compounds for adjuvant therapy are being anticipated as new strategies for the treatment of breast cancer using the cell cycle as a supportive point.

The PLK1 gene, which encodes a Ser/Thr protein kinase, is a member of the CDC5/Polo kinase subfamily. It exhibits high expression levels during mitosis, and its overexpression is a common characteristic in a variety of cancers (Yin et al., 2023). PLK1 directly or indirectly controls various events related to the cell cycle, such as controlling the maturation of centrosomes, activating CDK1 to regulate the transition of the cell cycle to the G2/M phase, participating in the orderly assembly of nodes-microtubules and stabilizing spindle assembly checkpoints, etc., and its overexpression is widely associated with tumorigenesis and poor prognosis is widely associated with tumorigenesis and poor prognosis (Kumar et al., 2016). both PLK1 and p53 are major players in various cellular events and are required to ensure normal cell cycle progression. A related study found that overexpression of PLK-1 can be mediated by interaction with the DNA-binding motif of p53 (Ando et al., 2004). However, despite the fact that the p53 gene is disrupted in more than 50% of human cancers, it remains questionable whether upregulation of PLK1 leads to downregulation of different types of p53 or vice versa.

The wild-type p53 gene is known as the “guardian of the cell”, which is able to detect DNA damage in cells and send out repair signals, or in the case of unrepairable damage, guide the cell towards programmed death, thus preventing the damaged cells from transforming into cancer cells. However, mutations in the p53 gene are found in more than 50% of human cancers. In addition to the loss of its oncogenic function, mutant p53 usually has an intrinsic, new oncogenic function, which is known as “gain of function”. It is worth noting that the frequency of p53 mutations in breast cancer is highly dependent on the subtype, with most hormone receptor-positive or luminal subtypes retaining a wild-type p53 status, whereas hormone receptor-negative patients predominantly carry p53 mutations with gain-of-function oncogenic activity, leading to a poorer prognosis. Relevant studies have shown that in hormone receptor-positive (HR+) breast cancer patients, although wild-type p53 deletion does not directly affect CDK4/6i activity or G1 phase arrest, it can promote tumor cells to re-enter the cell cycle and accelerate tumor progression through CDK2-mediated p130 phosphorylation, leading to resistance to CDK4/6 inhibitors (Kudo et al., 2024). Therefore, a two-pronged strategy targeting wild-type and mutant p53 in different subtypes of breast cancer may be of clinical relevance (Marvalim et al., 2023).

Steegmaier et al., 2007 reported the discovery of BI2536, a potent and selective small molecule inhibitor of Polo-like kinase 1 (PLK1). BI2536 is the first compound to induce all features associated with PLK1 inhibition, effectively inhibiting PLK1 enzyme activity at low nanomolar concentrations. This compound has been shown to cause mitotic arrest and induce apoptosis in human cancer cell lines from diverse tissue origins and tumor genomic backgrounds. Specifically, it causes cells to arrest at prometaphase, accumulate phosphohistone H3, and exhibit abnormal mitotic spindle formation (Steegmaier et al., 2007).

BI6727, an ATP-competitive dihydropteridinone-based kinase inhibitor with an IC50 of 0.87 nM, was developed based on the structure of BI2536. BI6727 selectively inhibits PLK1, inducing G2/M phase arrest and apoptosis in various tumor cells while causing reversible G1 and G2 phase arrest without apoptosis in normal cells (Rudolph et al., 2009). To date, BI6727 remains one of the most potent PLK1 inhibitors both *in vitro* and *in vivo*, demonstrating therapeutic potential in Phase I and II clinical trials and currently undergoing Phase III evaluation. Additionally, BI6727 has shown promising results when used in combination with other inhibitors (Gutteridge et al., 2016).

TP53 mutations, also known as tumor suppressor gene alterations, significantly impair the body’s natural anticancer mechanisms. When TP53 is inactivated, restoring its function becomes extremely challenging, as no other gene can effectively compensate for its loss. PC14586, a novel small-molecule p53 reactivator, selectively binds to a specific pocket in the TP53 Y220C mutant protein, aiming to restore the conformation and transcriptional activity of wild-type p53. Preclinical studies have demonstrated potent antitumor activity of PC14586 (Vu et al., 2025).

Despite ongoing challenges in the clinical application of PLK1 and p53 targeted therapies for breast cancer, BI2536 does not adversely affect cardiomyocytes but significantly impairs the proliferation of primary fibroblasts (Steegmaier et al., 2007). Due to epigenetic heterogeneity among patients, drugs effective in some may be ineffective in others. Similar to other anticancer agents, BI2536 and BI6727 have developed resistance (Solanes-Casado et al., 2021). PC14586 aims to restore p53 wild-type activity, primarily targeting TP53 Y220C mutations; however, since some breast cancers do not involve p53 mutations, its applicability is limited. In contrast, DHIE, a natural compound, can simultaneously modulate the PLK1-p53 signaling axis and demonstrate efficacy in both triple-negative and hormone receptor-positive breast cancers. Notably, breast cancer has not yet developed resistance to DHIE. Therefore, DHIE can be used either in combination with traditional compounds like taxol or as an alternative therapy.

A selectivity index (SI) ≥1.00 was considered effective, and a higher SI was considered safer. In this study, the SI of DHIE ranged from 3.56 to 3.79, indicating the potential of DHIE as a therapeutic candidate for breast cancer. However, there is a lack of toxicological analysis of DHIE in this study to determine the safe therapeutic dose and the maximum tolerated dose in human body. Clinical sample data are insufficient, and the possible adverse side effects of the drug are not further explored. This compound is still a certain distance from clinical application. Two different subtypes of breast cancer cell lines MDA-MB-231 and MCF-7 were used to examine whether DHIE exerts its effects through the PLK1-p53 signaling axis. We found that both drugs inhibited PLK1 expression, but to our surprise, one downregulated mutant p53 expression in triple-negative breast cancer cells (MDA-MB-231) and the other upregulated wild-type p53 expression in estrogen receptor-positive breast cancer cells (MCF-7), both of which led to CDK4 downregulation. Arrest the outcome of cell cycle and play a role in tumor suppression. In the future, our team will conduct lentivirus transfection mediated recovery of function tests to more accurately confirm that DHIE can inhibit PLK1, inhibit or reactivate different subtypes of p53, and arrest the cell cycle of breast cancer. The mechanism of how inhibition of PLK1 overexpression exerts regulatory effects on different types of p53 in different molecular subtypes of breast cancer and the differences in their outcomes deserve further exploration. Meanwhile, in addition to PLK1, the mechanism of action of other genes in the core gene cluster regulated by DHIE and their interactions with each other in breast cancer still deserve to be investigated. In addition, the effect of DHIE on breast cancer may not be limited to killing tumor cells *per se*, but also by influencing the infiltration of immune cells in the tumor microenvironment. We found immune infiltrating cells associated with the core gene clusters by CIBERSORT analysis, and the specific regulatory role of DHIE is still unclear, which is worth further investigation in the future, with a view to providing new options for clinical treatment.

## 5 Conclusion

**FIGURE 12:**
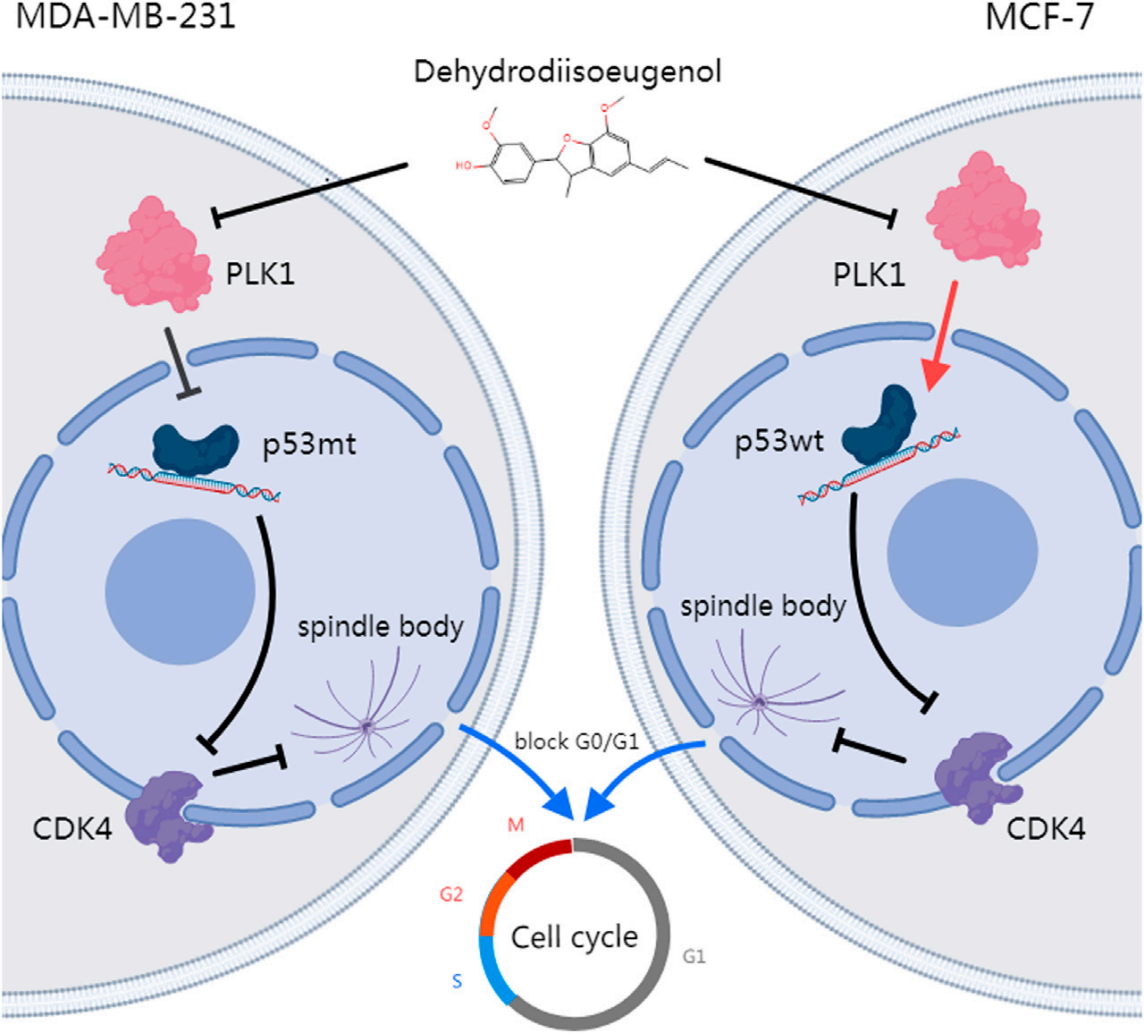
DHIE targets the PLK1-p53 axis to inhibit breast cancer cell cycle progression, with a synergistic effect observed when combined with DOX.

n this study, we utilized systematic pharmacological tools and bioinformatics to demonstrate that DHIE, the main active ingredient in AKH, inhibits breast cancer by suppressing the expression of PLK1 in breast cancer cells. This leads to the downregulation of mutant p53 (mtp53) or upregulation of wild-type p53 (wtp53), ultimately blocking the cell cycle at the G0/G1 phase (Figure 12). Therefore, our results suggest that DHIE can be used for the treatment of breast cancer, providing a new strategy for clinical use.

## Data Availability

The datasets presented in this study can be found in online repositories. The names of the repository/repositories and accession number(s) can be found in the article/Supplementary Material.

## Abbreviations

AKH, Alpinia Katsumadai Hayata; ANOVA, Analysis of variance; CDK4, Cell cycle protein-dependent kinase 4; DEGs, Differentially expressed genes; DHIE, dehydrodiisoeugenol; DissTOM, Dissimilarity topological overlap matrix; DL, Drug similarity; GEO, Gene Expression Omnibus; IARC, International Agency for Research on Cancer; MOD, Mean optical density; OB, Oral bioavailability; PBS:Phosphate-buffered saline PLK1, Polo-like Kinase 1; PPI, Protein-Protein interaction network; TCGA, The Cancer Genome Atlas; TOM, Topological overlap matrix; WGCNA, Weighted gene co-expression network analysis; WHO, World Health Organization.

## References

[B1] An J. Peng C. Tang H. Liu X. Peng F. (2021). New advances in the Research of Resistance to neoadjuvant chemotherapy in breast cancer. Int. J. Mol. Sci. 22 (17), 9644. 10.3390/ijms22179644 34502549 PMC8431789

[B2] An W. Zhang Y. Lai H. Zhang Y. Zhang H. Zhao G. (2022). Alpinia katsumadai Hayata induces growth inhibition and autophagy-related apoptosis by regulating the AMPK and Akt/mTOR/p70S6K signaling pathways in cancer cells. Oncol. Rep. 48 (2), 142. 10.3892/or.2022.8353 35730618 PMC9245070

[B3] Ando K. Ozaki T. Yamamoto H. Furuya K. Hosoda M. Hayashi S. (2004). Polo-like kinase 1 (Plk1) inhibits p53 function by physical interaction and phosphorylation. J. Biol. Chem. 279 (24), 25549–25561. 10.1074/jbc.M314182200 15024021

[B4] Ashokkumar K. Simal-Gandara J. Murugan M. Dhanya M. K. Pandian A. (2022). Nutmeg (Myristica fragrans Houtt.) essential oil: a review on its composition, biological, and pharmacological activities. Phytother. Res. 36 (7), 2839–2851. 10.1002/ptr.7491 35567294 PMC9541156

[B5] Atsumi T. Fujisawa S. Satoh K. Sakagami H. Iwakura I. Ueha T. (2000). Cytotoxicity and radical intensity of eugenol, isoeugenol or related dimers. Anticancer Res. 20 (4), 2519–2524. 10953321

[B6] Azizi E. Fouladdel S. Komeili Movahhed T. Modaresi F. Barzegar E. Ghahremani M. H. (2022). Quercetin effects on cell cycle arrest and apoptosis and doxorubicin activity in T47D cancer stem cells. Asian Pac J. Cancer Prev. 23 (12), 4145–4154. 10.31557/apjcp.2022.23.12.4145 36579996 PMC9971456

[B7] Bews H. J. Mackic L. Jassal D. S. (2024). Preventing broken hearts in women with breast cancer: a concise review on chemotherapy-mediated cardiotoxicity. Can. J. Physiol. Pharmacol. 102 (9), 487–497. 10.1139/cjpp-2023-0358 38039515

[B8] Bray F. Laversanne M. Sung H. Ferlay J. Siegel R. L. Soerjomataram I. (2024). Global cancer statistics 2022: GLOBOCAN estimates of incidence and mortality worldwide for 36 cancers in 185 countries. CA Cancer J. Clin. 74 (3), 229–263. 10.3322/caac.21834 38572751

[B9] Butt A. J. Caldon C. E. McNeil C. M. Swarbrick A. Musgrove E. A. Sutherland R. L. (2008). Cell cycle machinery: links with genesis and treatment of breast cancer. Adv. Exp. Med. Biol. 630, 189–205. 10.1007/978-0-387-78818-0_12 18637492

[B10] Chohan D. P. Biswas S. Wankhede M. Menon P. K A. Basha S. (2024). Assessing breast cancer through tumor microenvironment mapping of collagen and other biomolecule spectral Fingerprints─A review. ACS Sens. 9 (9), 4364–4379. 10.1021/acssensors.4c00585 39175278 PMC11443534

[B11] Desai D. Majrashi M. Pathak S. Almaghrabi M. Liu K. Pondugula S. R. (2024). Evaluate the *in vitro* effect of anthracycline and alkylating cytophosphane chemotherapeutics on dopaminergic neurons. Cancer Rep. Hob. 7 (4), e2074. 10.1002/cnr2.2074 PMC11021631 38627904

[B12] Godínez-Chaparro B. Pérez-Gutiérrez S. Pérez-Ramos J. Heyerdahl-Viau I. Hernández-Vázquez L. (2022). Synthesis and biological activities of dehydrodiisoeugenol: a review. Pharm. (Basel) 15 (11), 1351. 10.3390/ph15111351 PMC9694604 36355523

[B13] Gutteridge R. E. Ndiaye M. A. Liu X. Ahmad N. (2016). Plk1 inhibitors in cancer therapy: from laboratory to clinics. Mol. Cancer Ther. 15 (7), 1427–1435. 10.1158/1535-7163.Mct-15-0897 27330107 PMC4936921

[B14] Herzog S. K. Fuqua S. A. W. (2022). ESR1 mutations and therapeutic resistance in metastatic breast cancer: progress and remaining challenges. Br. J. Cancer 126 (2), 174–186. 10.1038/s41416-021-01564-x 34621045 PMC8770568

[B15] Hirakata M. Tomikawa E. Sakai C. Uchida M. Okano T. Shimozono R. (2024). TXB-001, a newly-developed polymer-conjugated anthracycline: significantly lower adverse effects in animal models of alopecia and hand-foot syndrome. Toxicol. Appl. Pharmacol. 485, 116912. 10.1016/j.taap.2024.116912 38521368

[B16] Iliaki S. Beyaert R. Afonina I. S. (2021). Polo-like kinase 1 (PLK1) signaling in cancer and beyond. Biochem. Pharmacol. 193, 114747. 10.1016/j.bcp.2021.114747 34454931

[B17] Khan M. M. Yalamarty S. S. K. Rajmalani B. A. Filipczak N. Torchilin V. P. (2024). Recent strategies to overcome breast cancer resistance. Crit. Rev. Oncol. Hematol. 197, 104351. 10.1016/j.critrevonc.2024.104351 38615873

[B18] Kudo R. Safonov A. Jones C. Moiso E. Dry J. R. Shao H. (2024). Long-term breast cancer response to CDK4/6 inhibition defined by TP53-mediated geroconversion. Cancer Cell 42 (11), 1919–1935.e9. 10.1016/j.ccell.2024.09.009 39393354 PMC12700659

[B19] Kumar S. Sharma A. R. Sharma G. Chakraborty C. Kim J. (2016). PLK-1: angel or devil for cell cycle progression. Biochim. Biophys. Acta 1865 (2), 190–203. 10.1016/j.bbcan.2016.02.003 26899266

[B20] Lee M. Y. Seo C. S. Lee J. A. Shin I. S. Kim S. J. Ha H. (2012). Alpinia katsumadai H(AYATA) seed extract inhibit LPS-induced inflammation by induction of heme oxygenase-1 in RAW264.7 cells. Inflammation 35 (2), 746–757. 10.1007/s10753-011-9370-0 21830094

[B21] Li C. Zhang K. Pan G. Ji H. Li C. Wang X. (2021). Dehydrodiisoeugenol inhibits colorectal cancer growth by endoplasmic reticulum stress-induced autophagic pathways. J. Exp. Clin. Cancer Res. 40 (1), 125. 10.1186/s13046-021-01915-9 33838688 PMC8035743

[B22] Liu D. You P. Luo Y. Yang M. Liu Y. (2018). Galangin induces apoptosis in MCF-7 human breast cancer cells through mitochondrial pathway and phosphatidylinositol 3-kinase/akt inhibition. Pharmacology 102 (1-2), 58–66. 10.1159/000489564 29879712

[B23] Marvalim C. Datta A. Lee S. C. (2023). Role of p53 in breast cancer progression: an insight into p53 targeted therapy. Theranostics 13 (4), 1421–1442. 10.7150/thno.81847 36923534 PMC10008729

[B24] Nam J. W. Seo E. K. (2012). Structural characterization and biological effects of constituents of the seeds of Alpinia katsumadai (Alpina Katsumadai Seed). Nat. Prod. Commun. 7 (6), 795–798. 10.1177/1934578x1200700626 22816310

[B25] Piezzo M. Cocco S. Caputo R. Cianniello D. Gioia G. D. Lauro V. D. (2020). Targeting cell cycle in breast cancer: CDK4/6 inhibitors. Int. J. Mol. Sci. 21 (18), 6479. 10.3390/ijms21186479 32899866 PMC7554788

[B26] Prasher P. Sharma M. (2024). Starch-based “smart” nanomicelles: potential delivery systems for doxorubicin. Drug Dev. Res. 85 (6), e22253. 10.1002/ddr.22253 39207174

[B27] Rudolph D. Steegmaier M. Hoffmann M. Grauert M. Baum A. Quant J. (2009). BI 6727, a Polo-like kinase inhibitor with improved pharmacokinetic profile and broad antitumor activity. Clin. Cancer Res. 15 (9), 3094–3102. 10.1158/1078-0432.Ccr-08-2445 19383823

[B28] Solanes-Casado S. Cebrián A. Rodríguez-Remírez M. Mahíllo I. García-García L. Río-Vilariño A. (2021). Overcoming PLK1 inhibitor resistance by targeting mevalonate pathway to impair AXL-TWIST axis in colorectal cancer. Biomed. Pharmacother. 144, 112347. 10.1016/j.biopha.2021.112347 34700228

[B29] Steegmaier M. Hoffmann M. Baum A. Lénárt P. Petronczki M. Krssák M. (2007). BI 2536, a potent and selective inhibitor of polo-like kinase 1, inhibits tumor growth *in vivo* . Curr. Biol. 17 (4), 316–322. 10.1016/j.cub.2006.12.037 17291758

[B30] Su Q. Wang J. Wu Q. Ullah A. Ghauri M. A. Sarwar A. (2021). Sanguinarine combats hypoxia-induced activation of EphB4 and HIF-1α pathways in breast cancer. Phytomedicine 84, 153503. 10.1016/j.phymed.2021.153503 33636580

[B31] Vu B. T. Dominique R. Fahr B. J. Li H. H. Fry D. C. Xu L. (2025). Discovery of rezatapopt (PC14586), a first-in-class, small-molecule reactivator of p53 Y220C mutant in development. ACS Med. Chem. Lett. 16 (1), 34–39. 10.1021/acsmedchemlett.4c00379 39811143 PMC11726359

[B32] Waks A. G. Winer E. P. (2019). Breast cancer treatment: a review. Jama 321 (3), 288–300. 10.1001/jama.2018.19323 30667505

[B33] Wang X. Q. Yang X. J. Li J. S. (2008). Studies on chemical constituents of alpinia katsumadai. Zhong Yao Cai 31 (6), 853–855. 18998568

[B34] Yin Q. Ma H. Bamunuarachchi G. Zheng X. Ma Y. (2023). Long non-coding RNAs, cell cycle, and human breast cancer. Hum. Gene Ther. 34 (11-12), 481–494. 10.1089/hum.2023.074 37243445 PMC10398747

[B35] Zhang J. Wang N. Zheng Y. Yang B. Wang S. Wang X. (2023). Naringenin in Si-Ni-San formula inhibits chronic psychological stress-induced breast cancer growth and metastasis by modulating estrogen metabolism through FXR/EST pathway. J. Adv. Res. 47, 189–207. 10.1016/j.jare.2022.06.006 35718080 PMC10173160

[B36] Zhang T. Guo S. Zhu X. Qiu J. Deng G. Qiu C. (2020). Alpinetin inhibits breast cancer growth by ROS/NF-κB/HIF-1α axis. J. Cell Mol. Med. 24 (15), 8430–8440. 10.1111/jcmm.15371 32562470 PMC7412407

